# Qualitätsmanagement in der Forschung – Die Sicht der Forschungsteams

**DOI:** 10.1007/s00103-026-04191-0

**Published:** 2026-02-10

**Authors:** Sophia Sohns, Janine Kleymann-Hilmes

**Affiliations:** Robert Koch-Institut, Forschungskoordination, Nordufer 20, 13353 Berlin, Deutschland

**Keywords:** Forschungslabore, Forschungsqualität, Mitarbeiterbefragung, Gute Wissenschaftliche Praxis, Ressourcenschonung, Research laboratories, Research quality, Employee surveys, Good scientific practice, Saving resources

## Abstract

**Einleitung:**

Forschende stehen Qualitätsmanagementsystemen (QMS) in Forschungslaboren gespalten gegenüber – es gibt Befürworter und Kritiker. Befürworter nutzen verschiedene Qualitätsmanagement-(QM-)Theorien und betonen Vorteile wie ressourcenschonenden Mehrwert. Kritiker hingegen zweifeln an Nutzen und Umsetzbarkeit. Ziel dieser Arbeit ist eine objektive Betrachtung von Vor- und Nachteilen eines QMS in Forschungslaboren anhand der Erfahrungen von Mitarbeitenden.

**Methoden:**

Das Robert Koch-Institut und Kooperationspartner führten ein QMS in Pilotforschungslaboren ein bzw. nutzten bestehende Systeme. Mitarbeitende wurden im Zeitraum von 2023 bis 2025 vor und nach der QMS-Einführung mit speziell dafür entwickelten Fragebögen befragt.

**Ergebnisse:**

Vorteile eines QMS sind verbesserte Dokumentation und Reproduzierbarkeit, optimierte Prozesse, gestärktes Qualitätsbewusstsein sowie bessere Zusammenarbeit. Dem stehen Nachteile wie höherer Zeit- und Arbeitsaufwand, Überregulierungsrisiko, mögliche Einschränkungen kreativer Freiräume, schwierige Verhaltensänderungen, begrenzter Einfluss auf Teamkultur und Gefahr von „Betriebsblindheit“ gegenüber. Bereits kurzfristig zeigten sich Veränderungen im Wissen, Handeln und in Einstellungen der Mitarbeitenden. Die Mehrheit befürwortet QM-Maßnahmen nach deren Erprobung und empfiehlt sie weiter.

**Diskussion:**

Forschungslabore benötigen flexible QMS, die auf individuelle Bedürfnisse eingehen. Maßnahmen sollten gemeinsam im Team entwickelt und gezielt dort eingesetzt werden, wo sie die Effizienz steigern. Entscheidend sind das Engagement aller Beteiligten und ein ausgewogenes Verhältnis von Aufwand und Nutzen, da übermäßige Regulierung hinderlich wirkt.

**Zusatzmaterial online:**

Zusätzliche Informationen sind in der Online-Version dieses Artikels (10.1007/s00103-026-04191-0) enthalten.

## Abstract (en)

**Introduction:**

Researchers are divided on quality management systems (QMS) in research laboratories—there are supporters and critics. Supporters use various quality management (QM) theories and emphasize advantages such as resource-saving added value. Critics, on the other hand, doubt their usefulness and feasibility. The aim of this study is to provide an objective assessment of the advantages and disadvantages of a QMS in research laboratories based on employee experiences.

**Methods:**

The Robert Koch Institute and its cooperation partners introduced a QMS in pilot research laboratories or used existing systems. Employees were surveyed between 2023 and 2025 before and after the QMS implementation using specially developed questionnaires.

**Results:**

The advantages of a QMS include improved documentation and reproducibility, optimized processes, increased quality awareness, and better collaboration. These are offset by disadvantages such as increased time and effort, the risk of overregulation, potential restrictions on creative freedom, difficult behavioral changes, limited influence on team culture, and the risk of “organizational blindness.” Changes in employee knowledge, actions, and attitudes were already evident in the short term. The majority support QM measures after testing them and recommend them.

**Discussion:**

Research laboratories require flexible QMS that respond to individual needs. Measures should be developed jointly within the team and implemented specifically where they increase efficiency. The commitment of all those involved and a balanced relationship between effort and benefit are crucial, as excessive regulation has a hindering effect.

## Einleitung

Qualitätsmanagementsysteme (QMS) gibt es in nahezu allen Branchen. Sie dienen der Lenkung und Sicherung von Ergebnisqualität sowie der kontinuierlichen Verbesserung. Während ein QMS in medizinischen Laboren zur Gewährleistung der Patientensicherheit verpflichtend ist [1, 2], ist die Anwendung in Forschungslaboren eine Rarität. In der Forschung existieren weder anerkannte Qualitätsstandards noch Zertifizierungs- oder Akkreditierungsverfahren, sodass sich die Organisation von Forschungsprozessen selbst innerhalb einer Einrichtung stark zwischen einzelnen Arbeitsgruppen unterscheiden kann. Ein Austausch über entsprechende *Best Practices* zwischen den Laboren findet nur selten statt. Die aktuelle Situation von Forschenden – geprägt von Erfolgs- und Zeitdruck, Personalfluktuation, Regulatorik sowie Konkurrenz [3–5] – in Verbindung mit der hohen Zahl nicht replizierbarer Veröffentlichungen [6] und der steigenden Publikationsrückzugsrate [7] macht eine Optimierung der Forschungsprozesse erforderlich, wie in den Publikationen [8] und [9] umfassend dargelegt wurde. Dementsprechend gibt es verschiedene Argumente von QM-Befürwortern, ein QMS in der Forschung einzuführen. Sie berichten von Vorteilen wie optimierten Forschungsprozessen, klaren Verantwortlichkeiten, Wissenserhalt und der Sicherung der Ergebnisqualität [10–12]. Demgegenüber stehen Forschende, die ein QMS eher kritisch betrachten. Ihre Argumente sind u. a. nicht notwendiger Formalismus bzw. erhöhter bürokratischer Aufwand und eine gefürchtete Einschränkung wissenschaftlicher Kreativität [8].

Aufgrund dieser unterschiedlichen Ansichten und der Tatsache, dass bislang kein QMS für Forschungslabore auf seinen Nutzen und die Akzeptanz hin überprüft wurde, wurde am Robert Koch-Institut (RKI) sowie bei Kooperationspartnern ein QMS in Pilot-Forschungslaboren erprobt. Ziel war es, herauszufinden, ob es tatsächlich den von den Befürwortern proklamierten positiven Einfluss haben kann oder ob es den Forschungsalltag, wie die Kritiker empfinden, behindert. Dafür wurde ein speziell für Forschungslabore entwickeltes QMS implementiert und vor und nach der Einführungsphase von ca. 10 Monaten durch Mitarbeiterbefragungen evaluiert. Die Ergebnisse dieser Untersuchung werden in dieser Originalarbeit vorgestellt und diskutiert. Angesichts der beschriebenen Situation in der biomedizinischen Forschungsgemeinschaft soll diese Arbeit Forschenden eine erste Orientierung zu den Vor- und Nachteilen eines QMS bieten und den Austausch über *Best Practices *in der Forschungsorganisation mit dem Ziel der Ressourcenschonung und Prozessoptimierung anregen.

## Methoden

### Entwicklung eines QMS für die Forschung.

Im Zeitraum von 2022 bis 2025 wurde ein praxisnahes QMS u. a. nach den Theorien der DIN EN ISO 9001 [13] speziell für das hier beschriebene Vorhaben entwickelt, mit den Forschungsleitenden zusammen optimiert und eingeführt. Die dafür definierten Anforderungen basieren auf internationalen Labornormen und der Guten Wissenschaftlichen Praxis (GWP) der Deutschen Forschungsgemeinschaft (DFG; [14]). Das QMS, seine Charakteristika und dessen Intention wurden in Publikation [9] detailliert beschrieben.

### Rekrutierung unterschiedlicher Forschungslabore.

Die Rekrutierung der Pilotlaborteams erfolgte über die jeweiligen Laborleitungen. Für das Projekt wurden mehrere Forschungslabore des RKI und einer Universität mit unterschiedlichen fachlichen Ausrichtungen, organisatorischen Strukturen und Personalausstattungen ausgewählt (Labore A–E). Die Pilotlabore forschen unabhängig voneinander in biologischen oder medizinischen Feldern und besitzen eigene Strukturen und Prozesse.

Überblick der teilnehmenden Labore und Rücklauf der Fragebögen:

- Labor A: 46 Mitarbeitende, starke Personalfluktuation, über 20 Nachwuchswissenschaftlerinnen und -wissenschaftler pro Jahr, Rücklauf: *Knowledge, Attitude and Practices-Befragung* 1 (KAP1) 50,0 %, KAP2 47,3 %,
- Labor B: 4 Mitarbeitende, Personalfluktuation, weniger als 10 Nachwuchswissenschaftlerinnen und -wissenschaftler pro Jahr, Rücklauf: KAP1 100 %, KAP2 50,0 %,
- Labor C: 4 Mitarbeitende, seit Jahren konstantes Team, Rücklauf: KAP1 und KAP2 100 %,
- Labor D: 23 Mitarbeitende, Personalfluktuation, weniger als 10 Nachwuchswissenschaftlerinnen und -wissenschaftler pro Jahr, Rücklauf: KAP1 65,2 %, KAP2 43,5 %,
- Labor E: 12 Mitarbeitende, Personalfluktuation, weniger als 10 Nachwuchswissenschaftlerinnen und -wissenschaftler pro Jahr, Rücklauf: KAP1 100 %, KAP2 66,7 %.

Als Vergleichsorganisation 1 (V1) wurden Labore einer Ressortforschungseinrichtung mit einem seit über 10 Jahren etablierten QMS in der Forschung ausgewählt. Dieses basiert auf der GWP [14] und weist Ähnlichkeiten zu dem der Pilotlabore auf. Es wurden 55 Personen befragt. Das Forschungsfeld umfasst vorwiegend chemische, physikalische und lebenswissenschaftliche Disziplinen.

Das Vergleichslabor 2 (V2) einer universitären Einrichtung verfügt über ebenso langjährige Erfahrung mit einem QMS in ihrer Forschung, das nach der ISO 9001 – *Qualitätsmanagementsysteme – Anforderungen* aufgebaut war. Nach einem Leitungswechsel vor über 2 Jahren wurde das QMS jedoch eingestellt und die Prozesse und Dokumente abgebaut. Befragt wurden 6 Personen, die aktiv mit dem QMS gearbeitet und es mitgestaltet hatten. Der Forschungsschwerpunkt liegt hier im medizinischen Bereich.

### Knowledge, Attitude and Practices (KAP) – Befragung.

Es wurden im Zeitraum von 2023 bis 2025 2 KAP-Befragungen in den Pilotlaboren (*n* = 5; A–E) durchgeführt, um Wissen, Einstellungen und Praktiken der Mitarbeitenden zur qualitätsgesicherten Forschung und zum QM zu erheben. Die erste Befragung (KAP1, s. Onlinematerial 1 – OM1) fand vor der QMS-Einführung statt, die zweite (KAP2, s. OM2) ca. 10 Monate später. Die Teilnahme war anonym und datenschutzrechtlich geprüft. Um Verzerrungen zu vermeiden, wurde die KAP1 nicht durch vorherige Infoveranstaltungen zum QM durch das Projektteam beeinflusst.

Auch in V1 und V2 wurde eine KAP-Befragung durchgeführt, um mögliche Unterschiede in den Befragungsergebnissen zwischen langjährigen Erfahrungen mit QMS oder eingestellten bzw. nicht vorhandenen QM-Strukturen zu erheben.

Befragt wurden alle Labormitarbeitenden mit direktem Forschungsbezug. Personen aus Verwaltung oder Management, die weder praktische Labortätigkeiten ausführen noch deren Durchführung betreuen, wurden nicht in die Befragung einbezogen, da diese auf die tägliche Laborarbeit ausgerichtet ist. Die Befragung wurde in regulären Teambesprechungen vorgestellt und während dieser von den Mitarbeitenden unabhängig voneinander auch ausgefüllt. Die Daten wurden nach Forschungslaboren sortiert erhoben.

### Statistik.

Zur statistischen Auswertung des Fragebogens wurden die Antworten gemäß der QM-Intention bewertet. Die Antwort mit der höchsten QM-Intention erhielt 1 Punkt, das Gegenteil −1. Bei mehreren Antwortmöglichkeiten erfolgte eine Abstufung: 1, 0,5, 0, −0,5, −1.

Beispiel: „Ich komme in unserem Ablagesystem schnell an die Dokumente bzw. Daten heran, die ich benötige.“

- Trifft nicht zu = −1
- Trifft eher nicht zu = −0,5
- Unentschieden = 0
- Trifft eher zu = 0,5
- Trifft zu = 1

Eine Normalverteilung wurde mithilfe des Shapiro-Wilk-Tests geprüft. Da keine vorlag, wurde der Mann-Whitney-Test zur Signifikanzbestimmung verwendet. Das Signifikanzniveau beträgt *p* < 0,05.

## Ergebnisse

### Veränderungen durch Einführung des Qualitätsmanagementsystems

Die Labore verfügten bereits vor Einführung des QMS in vielen Bereichen über Qualitätsbewusstsein und funktionierende Prozesse. In dem Untersuchungsintervall von ca. 10 Monaten konnten zwischen KAP1 und KAP2 nur in wenigen Bereichen signifikante Unterschiede festgestellt werden (Abb. 1). Dennoch sind bereits erste Gegensätze erkennbar. Da die Pilotlabore unterschiedliche Schwerpunkte bei der flexibel gestaltbaren QMS-Einführung setzten, spiegeln geringe oder fehlende Veränderungen nicht zwangsläufig eine Wirkung oder Wirkungslosigkeit des QMS wider, sondern vielmehr, dass der entsprechende Aspekt im jeweiligen Labor noch nicht im Fokus stand.

**Abb. 1:**
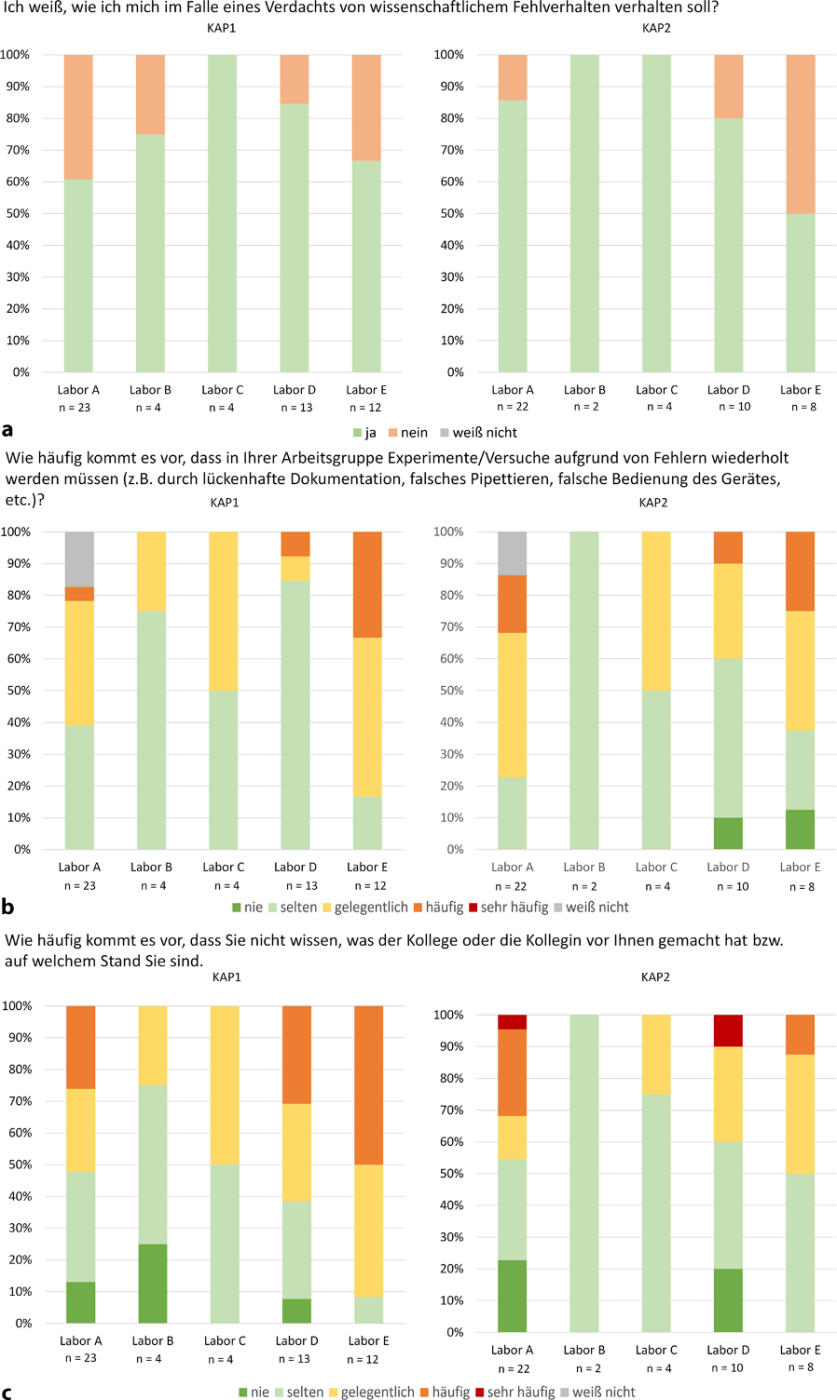
Vergleich der Knowledge, Attitude and Practices (KAP)-Befragung1- und KAP2-Befragung der Pilotlabore. Unterschiedliche Implementierungsschwerpunkte des Qualitätsmanagementsystems (QMS) erklären geringe oder fehlende Veränderungen; diese spiegeln nicht zwingend eine Wirkung oder Wirkungslosigkeit wider. **a** bis **e** zeigen eine Auswahl; weitere Abbildungen (Z1a–Z1g) sind im Onlinematerial 3 enthalten

**Abb. 1:**
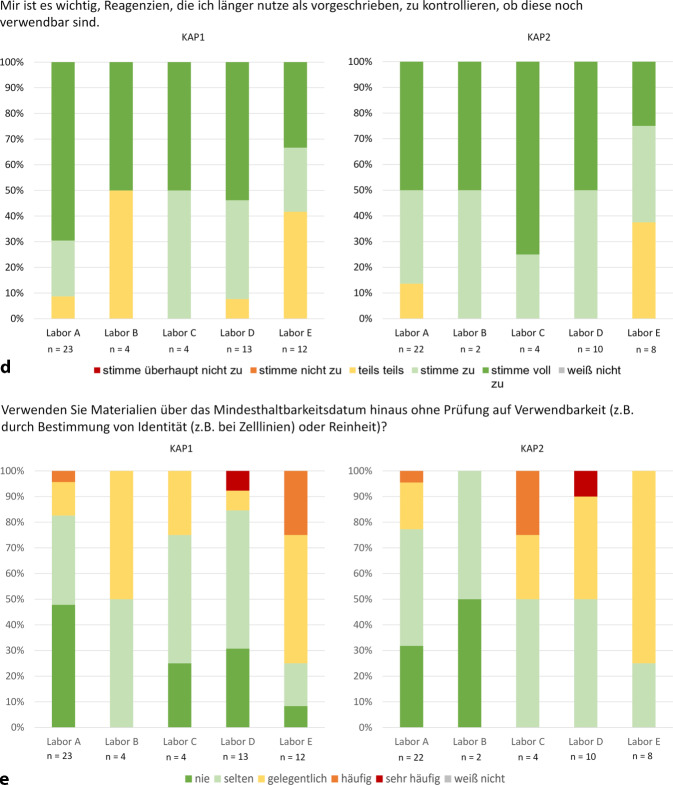
(Fortsetzung)

Die Antworten aus Vergleichslabor V2 (Abb. 2) weisen darauf hin, dass bei den Mitarbeitenden teilweise noch Kenntnisse aus der Zeit der Anwendung ISO-9001-zertifizierter Prozesse vorhanden waren. Sie zeigen aber auch, dass ohne kontinuierliche Pflege und ohne das Engagement der Leitung die QM-Strukturen zerfallen.

**Abb. 2:**
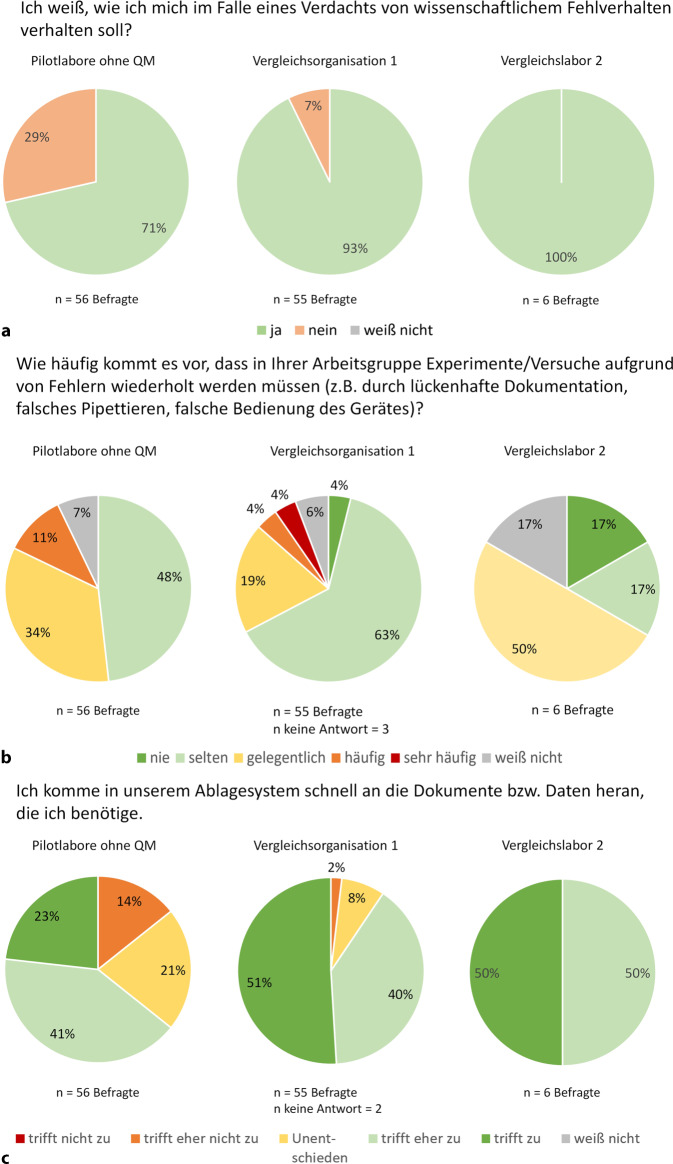
Vergleich der KAP1-Pilotlabore mit Vergleichsorganisation V1 und Vergleichslabor V2. V1 verfügt seit über 10 Jahren über ein Qualitätsmanagementsystem (QMS) in Forschungslaboren, während V2 das Forschungs-QMS nach 10 Jahren ungeordnet auflöste und damit einem Labor ohne formales QM entspricht, jedoch mit verbliebenen QM-relevanten Kenntnissen. Rundungsbedingte Abweichungen können zu geringfügigen Differenzen in den Grafiken führen. **a** bis **e** zeigen eine Auswahl; weitere Abbildungen (Z2a–Z2g) sind im Onlinematerial 3 dargestellt

**Abb. 2:**
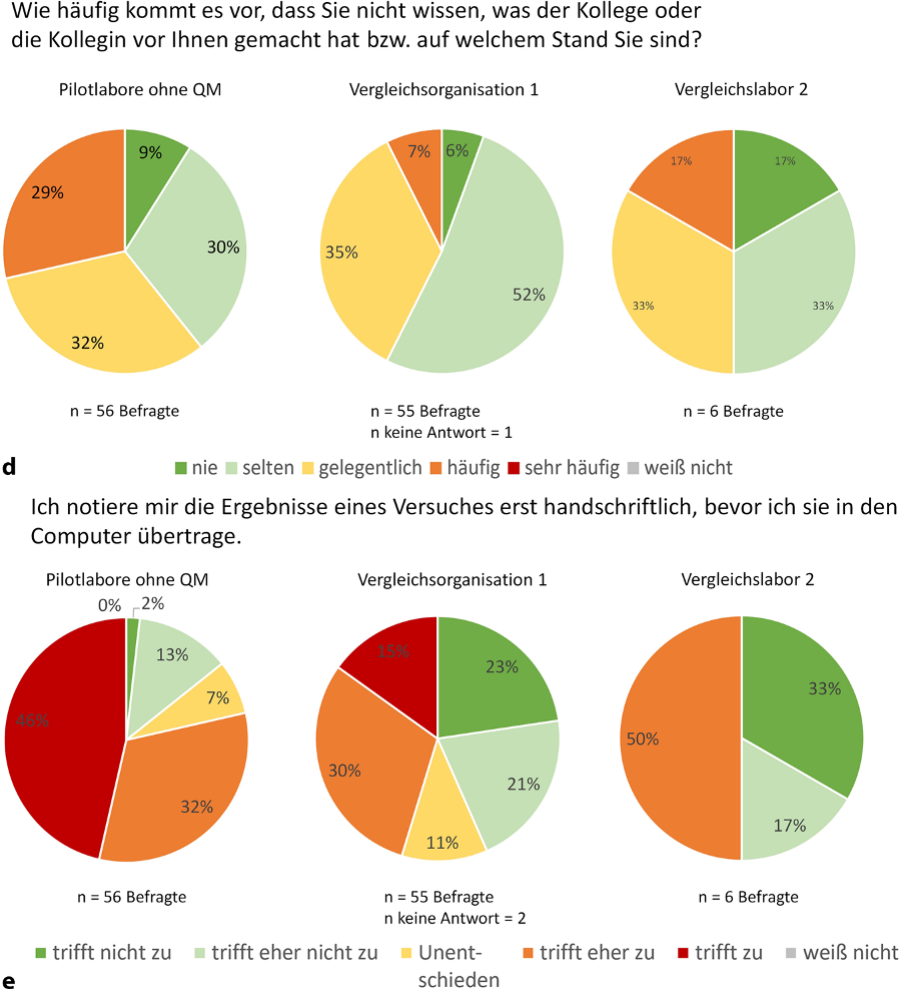
(Fortsetzung)

#### Stärken des Qualitätsmanagementsystems

Im Folgenden werden 3 bereits erkennbare Stärken des QMS erläutert: (1) systematische Identifikation und Verbesserung alltäglicher Herausforderungen, (2) die Stärkung des Bewusstseins für Prozesse und (3) die Verbesserung der Nachvollziehbarkeit.

##### Systematische Identifikation und Verbesserung alltäglicher Herausforderungen.

Das QM hat geholfen, bekannte, aber im Alltag oft vernachlässigte, unliebsame und dennoch wichtige Themen systematisch aufzudecken. Ein Beispiel ist die Verständlichkeit von Standardarbeitsanweisungen (SOPs), die nicht immer als gegeben angesehen werden kann. In der KAP1 bewerteten 30 % der Mitarbeitenden in Labor A, 15 % in D und 67 % in E die Verständlichkeit mit „teils, teils“ oder schlechter. In KAP2 konnte in Labor E bereits dieser Prozentsatz der Stimmen um 42 % reduziert werden, vorrangig zugunsten der Bewertung „stimme einer Unverständlichkeit (überhaupt) nicht zu“ (Prozentsatz KAP1: 33 %, KAP2: 63 %, Abb. Z1a im OM 3). Das QMS sorgt für einen systematischeren Umgang mit SOPs, einschließlich eines Mehr-Augen-Prinzips zur Prüfung auf Verständlichkeit und Anwendbarkeit.

Zudem wurden Unterschiede im Dokumentationsverhalten festgestellt. In V1 erfolgte die Dokumentation zügiger als in den Pilotlaboren (*p* = 0,0023). Während nur 11 % der V1-Mitarbeitenden ihre Daten nicht umgehend dokumentierten oder unentschieden waren, lag dieser Anteil in den Pilotlaboren bei 20 % (Abb. Z2a im OM 3). In der V1 werden Projektaufzeichnungen durch regelmäßige interne Audits auf Vollständigkeit und Nachvollziehbarkeit geprüft, wodurch Inkonsistenzen schneller erkannt und behoben werden können.

##### Stärkung des Bewusstseins für Prozesse.

Das QM hat dazu beigetragen, dass Organisations- und Laborprozesse, Dokumente und Kennzeichnungen bewusster wahrgenommen und geschätzt werden. In KAP1 zeigte sich, dass das Wissen im Labor hierzu stark variierte, was das Risiko unerwünschter Parallelprozesse erhöht.

Ein Beispiel ist die Kenntnis der Gerätebetreuenden innerhalb des Labors. Obwohl sie in allen Laboren existierten, waren sie in Labor B 75 %, in C 25 % und in A 9 % der Mitarbeitenden nicht bekannt. In KAP2 steigerte Labor C den Bekanntheitsgrad von verantwortlichen Personen auf 100 % (Abb. Z1b im OM 3).

Ein weiteres Beispiel betrifft die Protokollierungen von Teammeetings. Nicht alle wussten, dass Protokolle geführt wurden, während andere irrtümlich davon ausgingen. In Labor B wurden vor dem QM bereits Protokolle geführt, doch nur 25 % wussten davon. In Labor D hingegen glaubten 69 % fälschlicherweise an deren Existenz und obwohl nach KAP1 dort tatsächlich Protokolle eingeführt wurden, sank die Bekanntheitsrate in KAP2 sogar um 9 %, was auf eine unzureichende Prozesskommunikation hindeutet. Labor A führte erfolgreich Protokolle ein und informierte das Team darüber. Während in KAP1 nur 9 % der Mitarbeitenden angaben, dass Protokolle existieren, waren es in der KAP2 96 % (*p* < 0,0001, Abb. Z1c im OM 3). Auch in V1 war die Bekanntheit von Protokollen signifikant höher als in den Pilotlaboren ohne QM (*p* = 0,0011). 66 % der Mitarbeitenden waren informiert, in den Pilotlaboren nur 43 % (Abb. Z2b im OM 3).

Ähnliche Probleme traten bei Einarbeitungsplänen auf. Vor dem QM gaben die Forschungsleitungen in A, B und E an, dass solche Pläne existieren, doch waren sie nicht allen bekannt (A: 44 %, E: 17 %). In C und D hingegen glaubten Mitarbeitende fälschlicherweise an deren Existenz (C 50 %, D 15 %, Abb. Z1d im OM 3). Die V1 zeigte eine deutlich höhere Kenntnis über vorhandene Dokumente (*p* < 0,0001). 94 % bestätigten einen Einarbeitungsplan, während es in den Pilotlaboren nur 55 % waren (Abb. Z2c im OM 3).

Ein weiteres Beispiel für mangelnde Kenntnisse zu Prozessen ist die Bekanntheit einer Wartungsliste. In KAP1 kannten 52 % (A), 25 % (B), 25 % (C), 85 % (D) und 33 % (E) eine solche Übersicht, in A nahm die Unsicherheit in KAP2 zu. Die Zahl derjenigen, die von der Existenz einer Liste überzeugt waren, sank um 39 % (*p* = 0,0106), während „Weiß-nicht“-Antworten um 37,6 % zunahmen (Abb. Z1e im OM 3). Durch die Besprechung von Prozessen und Dokumenten wird die Existenz von Dokumenten nun hinterfragt.

Ähnliche Inkonsistenzen zeigten sich bei der Sichtbarkeit der Kalibrierstati. Während in B und D Kalibrierstati tatsächlich sichtbar waren und mindestens der Hälfte der Mitarbeitenden bekannt waren (B: 75 %, D: 50 %), nahmen in A (39 %), C (25 %) und E (33 %) viele irrtümlich an, dass eine Kennzeichnung existiert (Abb. Z1f im OM 3). Dies zeigt eine unzureichende Nutzung vorhandener Kennzeichnungen aufgrund von Unwissenheit oder angenommener mangelnder Relevanz.

Nicht nur die Bekanntheit von Teamabläufen variierte, sondern auch von GWP-relevanten Prozessen, besonders beim Umgang mit wissenschaftlichem Fehlverhalten. In KAP1 wussten 39 % in A nicht, wie sie sich in einem solchen Fall verhalten sollten. In KAP2 sank dieser Anteil aufgrund beginnender verpflichtender Schulungen auf 14 % (Abb. 1a). In V1 war die Kenntnis über das richtige Verhalten signifikant höher (*p* = 0,0056). 93 % der Mitarbeitenden waren informiert, da GWP-Inhalte dort regelmäßig obligatorisch geschult werden. In den Pilotlaboren lag dieser Anteil bei 71 %, in V2, in dem Kenntnisse noch aus QM-Zeiten vorhanden waren, bei 100 % (Abb. 2a). Trotz der GWP als Bestandteil des Berufsethos, bleiben regelmäßige Sensibilisierungen vorteilhaft.

Diese Beispiele zeigen, dass das Wissen über Prozesse uneinheitlich ist. Geordnete Abläufe, Dokumentationen und Kennzeichnungen erleichtern Tätigkeiten (v. a. bei seltenen Aufgaben), sichern Qualität und minimieren Risiken (z. B. durch Beschriftungen an speziellen Arbeitsplätzen). Zudem stellen sie sicher, dass alle Mitarbeitenden über dieselben Informationen verfügen, Zuständigkeiten klar sind und Ergebnisse rückverfolgbar bleiben.

Die positiven Effekte klarer Prozesse verdeutlicht V1. Dort mussten signifikant weniger Experimente aufgrund von Fehlern wie lückenhaften Aufzeichnungen, falschem Pipettieren oder fehlerhafter Gerätebedienung wiederholt werden als in den Pilotlaboren (*p* = 0,0421). Während in der V1 27 % der Mitarbeitenden „gelegentlich“ oder „(sehr) häufig“ Wiederholungen angaben, waren es in den Pilotlaboren 45 % (Abb. 2b). Labor E konnte sich hier bereits verbessern. Der Anteil von Angaben, dass häufig oder gelegentlich wiederholt werden müsse, sank von 83 % in KAP1 auf 63 % in KAP2 (Abb. 1b).

##### Verbesserung der Nachvollziehbarkeit.

Ein QMS trägt zur Strukturierung des Ablagesystems bei und fördert den Teamaustausch. Besonders in größeren Laboren gab es eine tendenzielle Unzufriedenheit mit dem Ablagesystem. E steigerte die Zufriedenheit zwischen den KAP-Befragungen von 25 % auf 50 % („trifft (eher) zu“, Abb. Z1g im OM 3). V1 war mit dem Ablagesystem signifikant zufriedener (*p* = 0,0002). 91 % fanden sich gut zurecht („trifft (eher) zu“), während es in den Pilotlaboren nur 64 % waren (Abb. 2c).

Auch der Kenntnisstand über die Tätigkeiten der Teammitglieder verbessert sich durch QM. V1 schnitt signifikant besser ab (*p* = 0,0284). Hier wussten 42 % gelegentlich oder häufig nicht, woran Kollegen arbeiten, im Vergleich zu 61 % in den Pilotlaboren ohne QM (Abb. 2d). C, D und E zeigten Verbesserungen zwischen den KAP-Befragungen, E sogar signifikant (*p* = 0,0380). In KAP1 hatten 50 % in C, 62 % in D und 92 % in E häufig oder gelegentlich keinen Überblick über die Tätigkeiten und Fortschritte. In KAP2 sank dieser Anteil um mindestens 20 % (C 0 %, D 40 %, E 51 %, Abb. 1c).

Diese Entwicklung zeigt, dass sich der Austausch und die Transparenz über Aufgaben und Prozesse durch Anwendung eines QMS verbessern können.

#### Schwächen des Qualitätsmanagementsystems

Das QM nicht alle Probleme löst, zeigt sich in den folgenden 3 Schwächen eines QMS: (1) keine automatische Verhaltensänderung, (2) Risiko „Betriebsblindheit“, (3) Änderung der Teamkultur ist komplex.

##### Keine automatische Verhaltensänderung.

Eine Verhaltensänderung tritt durch die Einführung eines QMS nicht automatisch ein – sie setzt die Bereitschaft zur Umstellung voraus. Das Projekt zeigte, dass die Veränderung von Gewohnheiten und Verhalten ohne intrinsische Motivation herausfordernd sein kann. So legten die Mitarbeitenden bspw. keine einheitliche Methode der Aufzeichnung fest, sondern nutzen mehrere parallel, am häufigsten das physische Laborbuch. In D und E existierte zudem ein elektronisches Laborbuch (ELB). In E arbeiteten 92 % damit, wobei 33 % gleichzeitig ein physisches Laborbuch führten. In D nutzen alle ein physisches Laborbuch, wobei 46 % zusätzlich ein ELB führten. Auch in V2, wo das ELB seit Langem etabliert ist, zeigte sich dieser Trend. Die parallele Nutzung ergibt sich aus der gewohnten und bevorzugten Aufzeichnungsart, die trotz neuer Hilfsmittel beibehalten wird, solange keine eigene Motivation zum Wechsel besteht. Dies führt zu „doppelter Buchführung“. Viele Mitarbeitende übertrugen Daten manuell vom Papier in den Computer – ein zusätzlicher Arbeitsschritt, der Zeit kostet und das Risiko von Datenverlusten oder Fehlangaben erhöht.

Nur V1, die kürzlich ein ELB einführte, schnitt hier signifikant besser ab (*p* < 0,0001). 45 % dokumentierten zunächst handschriftlich und übertrugen die Daten dann digital, verglichen mit 78 % in den Pilotlaboren (Abb. 2e). Auch in V1 blieben persönliche Gewohnheiten schwer zu ändern. So schnitt sie bei der Nachvollziehbarkeit von SOP-Abweichungen signifikant schlechter ab als die Pilotlabore (*p* = 0,0285). Während 25 % der V1 angaben, dass Änderungen nicht oder nur bedingt nachvollziehbar dokumentiert würden („unentschieden“ oder „trifft (eher) nicht zu“), lag dieser Anteil in den Pilotlaboren bei lediglich 8 % (Abb. Z2d im OM 3).

In D zeigte sich auch, dass persönliche Verhaltensweisen schwer zu ändern sind, besonders wenn sie von externen Faktoren, wie Zeitdruck, beeinflussbar sind. In KAP2 stimmten 100 % der Aussage (voll) zu, dass die Prüfung der Verwendbarkeit abgelaufener Materialien, die länger genutzt werden als vorgeschrieben, wichtig ist (Abb. 1d), doch nur 50 % setzten dies um. Zudem stieg die Nutzung abgelaufener Materialien ohne vorherige Prüfung signifikant an (D KAP1: 85 % nie oder selten ohne Prüfung, KAP2: 50 %; *p* = 0,0338, Abb. 1e). Zugleich gaben 62 % an, gelegentlich oder (sehr) häufig unter Zeitdruck zu stehen, was dieses widersprüchliche Verhalten beeinflussen könnte.

Das QMS hatte insgesamt nur wenig Einfluss auf die Einstellung der Mitarbeitenden zur Materialprüfung. Während A und C abgelaufene Materialien meist testeten (A: 83 %, C 75 % nie oder selten ohne Prüfung), prüften B und E seltener (B: 50 %, E: 25 % nie oder selten ohne Prüfung, Abb. 1e). Im Gegensatz zu D räumten sie dem aber auch die entsprechende Bedeutung ein (KAP1, A: 92 %, B: 50 %, C: 100 %, E: 58 % stimmten der Wichtigkeit einer Prüfung (voll) zu, Abb. 1d). Auch in diesen Laboren könnten äußere Faktoren, wie Zeitdruck, die Haltung mit beeinflussen. Labore mit weniger Zeitdruck (A: 39 %, C 25 % gelegentlich oder (sehr) häufig Zeitdruck) prüften Materialien häufiger und maßen der Kontrolle mehr Bedeutung bei als Labore mit höherem Zeitdruck (B 75 %, E 49 % gelegentlich oder (sehr) häufig Zeitdruck).

Ein QMS kann äußere Faktoren wie Zeitdruck nicht beseitigen, aber Prozesse und Übergaben optimieren, um Ressourcen zu schonen, Zeitverluste auszugleichen und das Risiko möglicher Fehler zu minimieren.

##### Risiko „Betriebsblindheit“.

Funktionierende Prozesse können dazu führen, dass das Bewusstsein für ihre Hintergründe und potenzielle Fehlerquellen mit der Zeit abnimmt. Dies zeigte sich in der V1 beim Umgang mit Materialien. Zwar testen die Mitarbeitenden abgelaufene Reagenzien vor der Nutzung häufiger als die Pilotlabore (93 % vs. 68 % nie oder selten ohne Prüfung, *p* < 0,0001, Abb. Z2e im OM 3), sie maßen dieser Kontrolle jedoch eine geringere Bedeutung bei (61 % vs. 82 % stimmten (voll) zu, dass es notwendig sei, *p* = 0,0486, Abb. Z2f im OM 3).

Dies verdeutlicht eine potenzielle Schwachstelle eines QMS. Regelmäßige Sensibilisierung und Austausch sind daher immer essenziell.

##### Änderung der Teamkultur ist komplex.

Ein QM kann Fehler als Chance zur Verbesserung nutzen, erfordert jedoch eine offene Fehlerkultur und gegenseitiges Vertrauen. Ein QMS allein kann die Teamkultur nicht verändern, da diese von Erfahrungen, Teamdynamik und Führungsstil geprägt ist.

So nahm beispielsweise V1 defekte Geräte seltener sichtbar außer Betrieb als die Pilotlabore (80 % vs. 94 % (sehr) häufig außer Betrieb genommen, *p* = 0,0276, Abb. Z2g im OM 2).

Zudem ist es essenziell, dass Laborleitende die Stimmung im Team regelmäßig reflektieren. So gaben alle Laborleitenden an, eine offene Fehlerkultur zu pflegen und dass Fehler mündlich kommuniziert würden. Dennoch befürworteten die Mitarbeitenden mehrheitlich ein anonymes Fehlermeldesystem („gelegentlich“ und „(sehr) häufig“ besteht der Wunsch: A 69 %, B 100 %, C 75 %, D 39 % und E 91 %).

### Einstellungen zu QM

Ein QMS entfaltet seine volle Wirkung erst, wenn es sowohl praktikabel ist als auch akzeptiert wird. Im Folgenden werden die Erfahrungen der Mitarbeitenden damit betrachtet.

Insgesamt sahen 83 % der Mitarbeitenden in den Pilotlaboren Verbesserungen durch das QM, insbesondere in der Dokumentation, Datennutzung, -sicherung und Ablage. Auch überarbeitete SOPs und ein besserer Informationszugang wurden positiv bewertet (Abb. Z3a im OM 3).

Da das QMS in V1 bereits lange bestand, kannten viele Beteiligte den vorherigen Zustand nicht. Dennoch nahmen 50 % Verbesserungen wahr, v. a. in der Dokumentationsvollständigkeit und Nachvollziehbarkeit. Positiv bewertet wurden optimierte Ablagesysteme, eine bessere Datenreproduzierbarkeit, erhöhte Sensibilisierung für Forschungsqualität und Datenmanagement sowie transparentere Forschungsprozesse (Abb. Z3b im OM 3).

In V2 berichteten 67 % der Mitarbeitenden von Verbesserungen, insbesondere durch klare Verantwortlichkeiten, optimierte Aufzeichnungen und Einarbeitungen, eine bessere Übersicht über Geräte und Chemikalien sowie Verbesserungen bei ELB und SOPs.

Neben den festgestellten Verbesserungen wurden die Mitarbeitenden auch zu Verschlechterungen befragt. 37 % der Pilotlabormitarbeitenden nahmen eine solche wahr, hauptsächlich durch den gestiegenen Zeitaufwand und mehr Arbeit (Abb. Z3c im OM 3). In V1 äußerten 35 % der Mitarbeitenden Kritik, insbesondere am erhöhten Arbeitsaufwand und an der Bürokratie, gefolgt vom Zeitaufwand (Abb. Z3d im OM 3). In V2 empfanden 17 % der Befragten manches als zu bürokratisch.

**Abb. 3:**
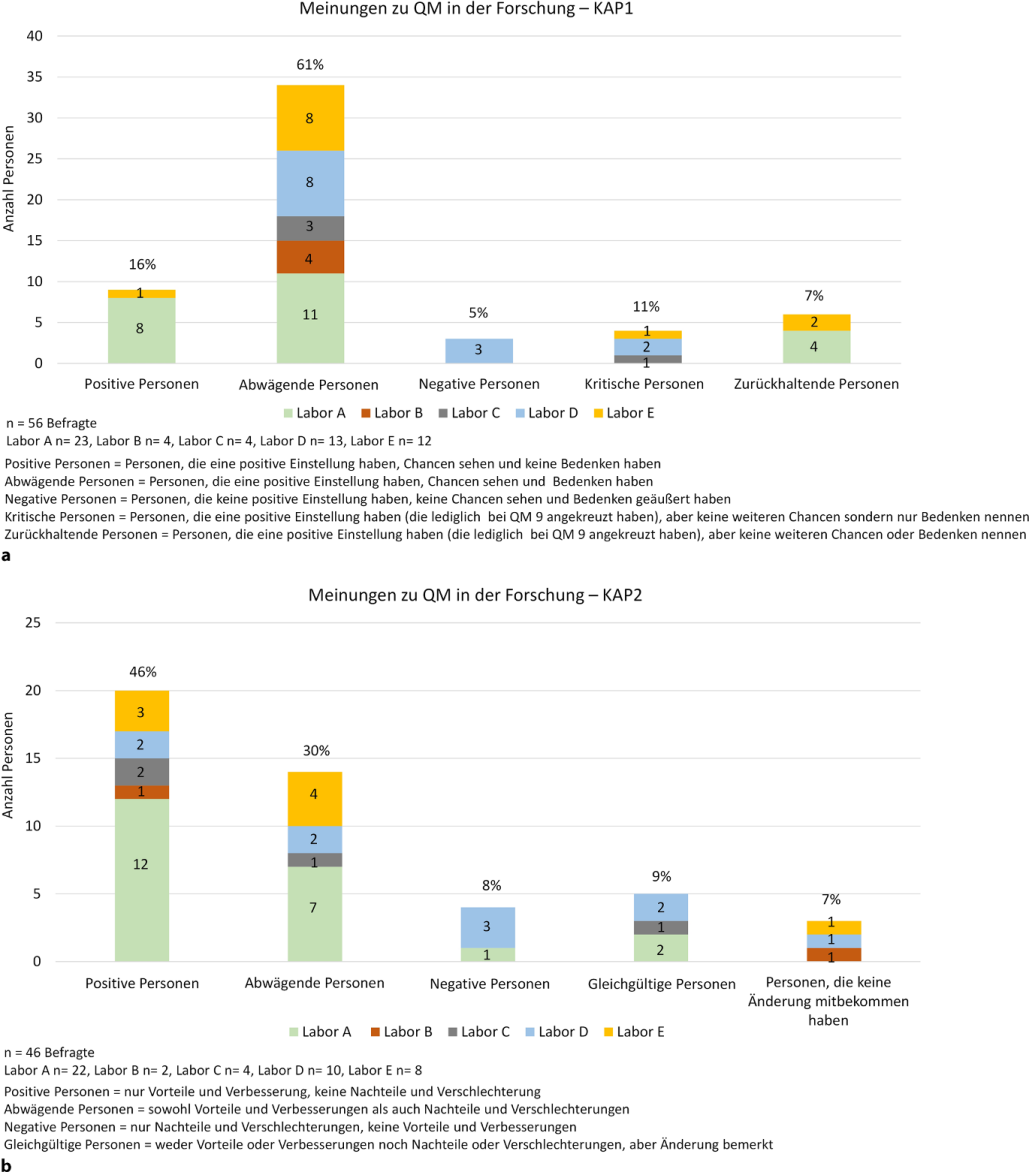
Meinungsverteilung zu Qualitätsmanagement (QM) in der Forschung; **a** Meinungsverteilung zu QM in der Forschung KAP1; **b** Meinungsverteilung zu QM in der Forschung KAP2

Die persönliche Haltung bzw. Offenheit gegenüber dem QMS ist maßgeblich für dessen Akzeptanz verantwortlich. Die Einstellung der Mitarbeitenden zum QM hat sich mit der Einführung der QM-Maßnahmen im Vergleich zu den vorherigen Erwartungen grundsätzlich verbessert (Abb. 3a, b).

In der KAP1 standen noch 61 % dem System abwägend gegenüber, während in der KAP2 dieser Anteil auf 30 % sank. Im Gegensatz dazu war bei gegenüber QM positiv eingestellten Personen eine fast dreifache Akzeptanzzunahme zu verzeichnen (KAP1 16 %, KAP2 46 %). Allerdings nahm auch der Anteil der negativ eingestellten Personen leicht zu (negativ KAP1: 5 %; negativ KAP2: 9 %, wobei 4 % QM-Maßnahmen aufgrund der GWP ablehnen).

**Abb. 4:**
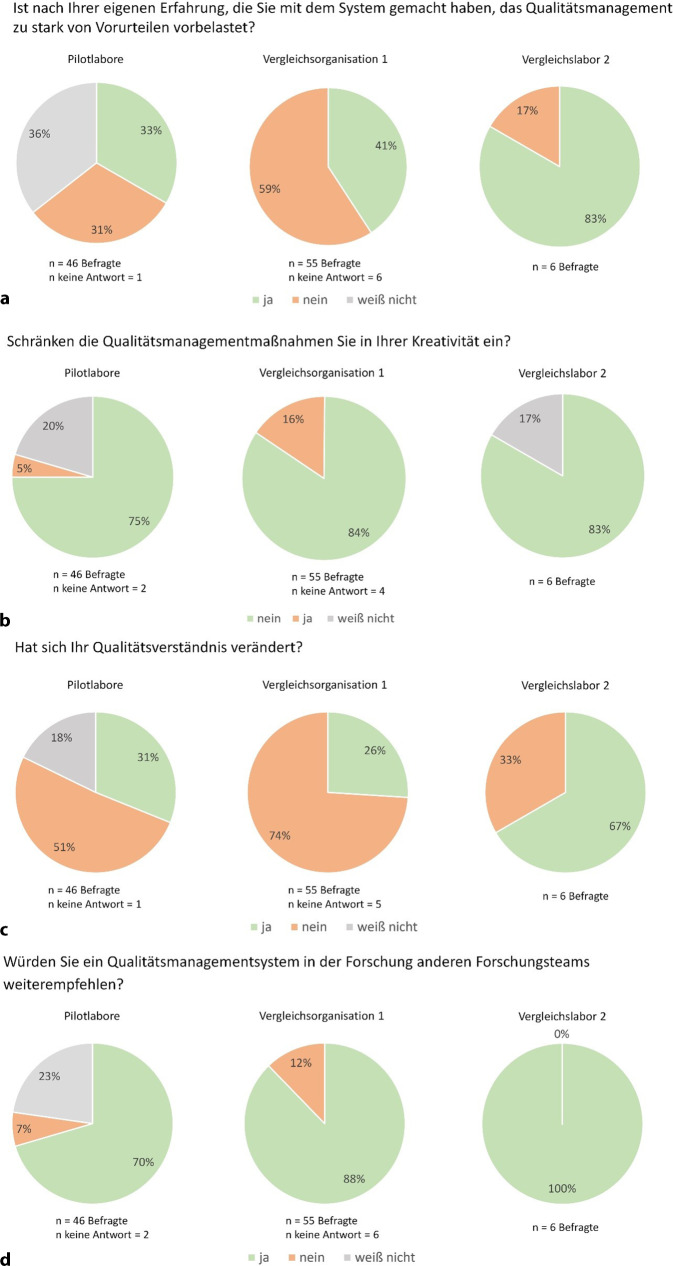
Vergleich der Meinungen zu Qualitätsmanagement (QM) zwischen KAP2-Pilotlaboren und Vergleichsorganisation V1 und Vergleichslabor V2

Die QM-Maßnahmen haben einen überwiegend positiven Eindruck hinterlassen, auch wenn viele ihre Vorurteile bestätigt sahen (Abb. 4a). 31 % der Pilotlabore und 59 % der V1 halten die QM-Vorurteile für berechtigt, während nur 17 % der V2 diesem zustimmen. Die Pilotlabore sind zudem aufgrund der vergleichsweise geringen Laufzeit des QM noch unsicher, was Vorurteile betrifft. Bei der Rechtfertigung von QM-Vorurteilen spielen die technische Umsetzung und Kommunikation des QM innerhalb der jeweiligen Organisation eine entscheidende Rolle. Ein häufiges Vorurteil, dass QM die Kreativität der Forschenden einschränke, bestätigte sich jedoch kaum (Abb. 4b). 75 % der Pilotlabormitarbeitenden verneinten eine Einschränkung, 21 % konnten dies aufgrund der kurzen Laufzeit noch nicht beurteilen. In V1 verneinten 84 % und in V2 83 % diese Annahme. Die Mitarbeitenden, die ihre Kreativität beeinträchtigt sahen, führten dies v. a. auf Zeitmangel und zusätzliche Aufgaben zurück, die als unpassend empfunden wurden. Darüber hinaus dokumentierte jemand, dass stupide Dokumentationstätigkeit die Kreativität „töten“ würde.

Bereits nach kurzer Zeit änderte sich das Qualitätsverständnis bei 31 % der Befragten der Pilotlabore (Abb. 4c). Angeführt wurde u. a. der erkannte Nutzen von Vorlagen für Dokumentation und Planung, ein besseres Prozessverständnis und gegenwärtige Überlegungen zur Verbesserung von Abläufen. Das Team erkannte, dass viele Prozesse bereits existierten, jedoch uneinheitlich umgesetzt wurden. Zudem wuchs das Bewusstsein für verständliche und replizierbare Ergebnisaufzeichnungen, die auch für Dritte nachvollziehbar sein müssen, was eine kritischere Betrachtung förderte. Auch stellten Teilnehmende, die anfängliche Bedenken zu Arbeitsaufwand, Kreativitätseinbußen und Zeitverlust geäußert hatten, fest, dass sich dies nicht bewahrheitet hat. Die Bedeutung von Qualität und der Nutzen des QM wurden erkannt, was auch von V1 bestätigt wurde. Dort gaben aufgrund der langen Laufzeit des QM nur 26 % ein verändertes Qualitätsverständnis an. Sie betonten, dass Forschung mehr als nur Messungen umfasst und durch QM effizienter sowie reproduzierbarer wird, sodass Projekte trotz Abwesenheit weitergeführt werden können. Hingegen erlebten in V2 alle den Vorher-nachher-Zustand. Zwei Drittel berichteten von einem veränderten Qualitätsverständnis. Achtsamere Arbeitsweisen, nachvollziehbarere Dokumentation und ein besseres GWP-Verständnis führten zu höherer Reproduzierbarkeit. Zudem wurde erkannt, dass SOPs und optimierte Abläufe die Forschungsqualität steigern.

Trotz des zum Teil zusätzlichen Arbeits- und Zeitaufwandes bei der Einführung eines QMS und der Bestätigung von QM-Vorurteilen empfehlen die meisten Befragten ein QM auch anderen Forschungseinrichtungen weiter (Abb. 4d): 71 % in den Pilotlaboren, 88 % in der V1 und 100 % im V2.

## Diskussion

Ein QMS bietet eine Chance für Forschungslabore, muss jedoch praxisnah gestaltet und stabil in den Arbeitsalltag integriert werden. Entscheidend ist, dass das Team den Nutzen erkennt und nicht nur auf Anweisung handelt. Daher sollten Mitarbeitende in den Gestaltungsprozess eingebunden werden, in dem das QM den Rahmen vorgibt, aber teamgerecht ausgestaltet wird.

Neben der Akzeptanz des Teams ist auch das Engagement der Führungskraft entscheidend, wie in V2 deutlich wurde. Aufgrund einer anderen Priorisierung scheiterte dort das QMS. Im Nachhinein können jedoch aus diesem Ablauf wichtige Erkenntnisse resümiert werden. Eine Überregulation führte zu übermäßiger Bürokratie (z. B. SOPs über SOPs, unnötige Formulare) und exzessives Auditverfahren. Theoretische Konzepte wie Leitbilder und Visionen wurden diskutiert, obwohl sie für viele Mitarbeitende wenig praxisrelevant waren. Zudem wurde das System auch kritischen Laborleitenden aufgezwungen, was zu Widerstand und ineffizienten Prozessen führte. Sinnvoller wäre es, mit interessierten Führungskräften zu starten und später mit dem erprobten System auf die kritischen Stimmen zuzugehen.

Im Verlauf des Projekts konnten sowohl Veränderungen im Wissen und in den Praktiken als auch in der Einstellung der Mitarbeitenden zu QM verzeichnet werden. Dadurch wurden Chancen und Risiken eines QMS in der Forschung sichtbar (Tab. 1). Ein gut abgestimmtes QM kann Abläufe, Qualität und Dokumentation im Labor verbessern.

**Tab. 1:** Chancen und Risiken eines Qualitätsmanagementsystems (QMS) in der Forschung

		Details	Verweise
*Chancen*	Verbesserte Aufzeichnungen und Dokumentationen	Das QM führte zu verbesserten Aufzeichnungen und einer präziseren Dokumentation. Die Vollständigkeit und Nachvollziehbarkeit wurden verbessert. Zudem hat es zu einer Optimierung im Umgang mit Daten und deren Sicherung beigetragen	Abb. Z1g Labor E, Z2a, 2c, Z3a, Z3b
	Optimierung von Prozessen	Es wurde festgestellt, dass die Kenntnis der Laborprozesse im Team variierte. Durch die Einführung des QM wurde das Bewusstsein für diese Prozesse geschärft. Es wurde angegeben, dass sich ein besseres Prozessverständnis entwickelte und die Arbeitsprozesse nun klarer strukturiert seien. Dies minimiert das Risiko von Parallelprozessen und Missverständnissen. Die Laborarbeit wird dadurch effizienter und nachvollziehbarer	Abb. 1b Labor E, Z2c, 2b, Z3a, Z3b
	Verbesserung der Reproduzierbarkeit von Forschung	Nachvollziehbarere und transparente Forschungsprozesse, optimierte Aufzeichnungen und Ablagesysteme sowie ein strukturierter Umgang mit Chemikalien, Verbrauchsmaterial und Geräten bilden die Grundlage für reproduzierbare und verlässliche Projekte	Abb. Z3a, Z3b
	Sensibilisierung für Qualität	Die Mitarbeitenden berichteten von einem gesteigerten Bewusstsein für die Bedeutung von Qualität in Forschungsprozessen. Bei mehreren hat sich das Verständnis von Qualität bereits weiterentwickelt. Auch ein wesentlicher Bestandteil eines Forschungs-QMS ist die regelmäßige Schulung der GWP als allgemeingültige Grundlage des wissenschaftlichen Arbeitens. Durch die Einführung des QMS und die damit verbundenen Schulungen verbesserte sich das Wissen über GWP-relevante Vorgänge	Abb. 1a Labor A, 2a, Z3a, Z3b, 4c
	Bessere Zusammenarbeit und Kommunikation	Das QM förderte die Zusammenarbeit und Kommunikation im Team. Es wurden zusammen mögliche Verbesserungsbereiche identifiziert und gemeinsam umgesetzt. Durch die Protokollierungen blieben auch abwesende Teammitglieder stets auf dem neusten Stand. Auch waren die Tätigkeiten der anderen Teammitglieder bekannter	Abb. Z1c Labor A, 1c Labor C, D und E, Z2b, 2d, Z3a
	Erhöhte Transparenz	Durch die Erstellung und Optimierung von SOPs sowie den gemeinsamen Austausch über Abläufe wurden viele Prozesse transparenter. Zudem hat sich das Wissen um die Tätigkeiten der Teammitglieder verbessert. Ein geeignetes Ablagesystem ermöglicht einen schnellen und unkomplizierten Zugriff auf relevante Informationen	Abb. Z1a Labor E, Z3a, Z3b
	Akzeptanz und Empfehlung	Die Mehrheit der Mitarbeitenden empfiehlt das QMS weiter. Zudem hat sich ihre Einstellung gegenüber dem System positiv verändert	Abb. 3 und 4d
*Risiken*	Erhöhter Zeit- und Arbeitsaufwand	Ein häufig genannter Nachteil war der hohe Zeit- und Arbeitsaufwand, der insbesondere in der Anfangsphase der QMS-Einführung spürbar war. Die Anpassung der bestehenden Prozesse, die Erstellung von SOPs, die Schulung der Mitarbeitenden sowie die Dokumentation der Abläufe erforderten Ressourcen und Zeit	Abb. Z3c, Z3d
	Gefahr der Überregulierung und Bürokratie	Ein QMS in der Forschung sollte eine ausgewogene Kosten-Nutzen-Relation aufweisen. Vorgabedokumente und Regulierungen sollten nur in dem Maße eingeführt werden, wie sie im Arbeitsalltag praktikabel sind. Dabei ist darauf zu achten, dass das System sich nicht aufbläht und letztlich durch umständliche Formulare, aufwendige Unterschriftenwege oder eine übermäßige Anzahl an SOPs an Effizienz verliert. Das System muss auch bei personellen Veränderungen weiterhin funktionsfähig und nachhaltig bestehen bleiben	Abb. Z3d, Erfahrung V2
	Mögliche Einschränkung der Kreativität	Einige Mitarbeitende, wenngleich weniger als ein Viertel, empfanden das QM als hinderlich für die kreative Arbeit. Dies führten sie insbesondere auf den erhöhten Zeitaufwand und die zusätzlichen administrativen Aufgaben zurück	Abb. 4b
	Widerstand und Blockaden durch Führungskräfte und Mitarbeitende	Im V2 führte die fehlende Unterstützung der Führungskräfte zu Blockaden und einer Verkomplizierung der Abläufe des QMS. Das System wurde hier teils den Leitungen aufgezwungen. Ständige Erinnerungen und Nacharbeiten führten zu Einbußen von Zeit und Ressourcen	Erfahrung V2
	Persönliche Verhaltensweisen schwierig zu ändern	Ein QM kann persönliche Verhaltensweisen, wie das Dokumentationsverhalten im Laborbuch oder die Verwendung von abgelaufenen Materialien, ohne intrinsische Motivation nur schwer verändern	Abb. 1d Labor A und E, 1e Labor A, D und E, Z2d
	Ein QM beeinflusst nicht die Teamkultur	Eine Teamkultur wird durch verschiedene Faktoren geprägt und basiert auf zwischenmenschlichen Erfahrungen. Sie kann nicht einfach durch die Einführung eines QM geändert werden	Abb. Z2g
	Risiko der Betriebsblindheit	Das Abarbeiten von SOPs kann zu Betriebsblindheit und dem Fehlen von Plausibilitätschecks führen. Auch trotz Durchführung eines festgelegten Verfahrens muss weiterhin auf die Sinnhaftigkeit von Ergebnissen und Prozessen geachtet werden	Abb. Z2e und f

Gleichzeitig sind aber auch die Herausforderungen zu bedenken, insbesondere der erhöhte Zeit- und Arbeitsaufwand bei der Einführung. In dieser Phase werden bestehende Prozesse betrachtet und optimiert, SOPs und Formblätter erstellt, Strukturen neu geordnet sowie Schulungen durchgeführt. Auch langfristig berichteten Labore mit etabliertem QM von erhöhtem Dokumentationsaufwand. Doch gerade dieser kann im Nachhinein die korrekte Methoden- und Ergebnisbeschreibung in einer Publikation, die Übergabe von Projekten und die Nachvollziehbarkeit auch noch Jahre später erleichtern. Auch durch die in der Regel hohe Personalfluktuation der Forschungslabore durch befristete Arbeitsverträge können eine unzureichende Dokumentation und Wissensübergabe zu zusätzlichen Kosten und Verzögerungen durch Nacharbeit führen. Auch regelmäßig wiederkehrende Fehler binden Ressourcen. Ein QM wirkt hier präventiv. Zwar lassen sich nicht alle Probleme vermeiden, doch ein gut implementiertes QMS minimiert Risiken und erhöht die Effizienz. Zukünftig erscheint der Einsatz künstlicher Intelligenz (KI) als Unterstützung im Labormanagement denkbar, um das QM in der akademischen Forschung zu rationalisieren. Die KI könnte Routineaufgaben übernehmen, wie das Erfassen, Versionieren und Archivieren von Laborprotokollen, die Sicherstellung der Nachvollziehbarkeit durch Audit-Trails sowie die Überprüfung der Konformität mit formalen Anforderungen. Darüber hinaus könnte sie Laborberichte, SOPs und Projektdaten verknüpfen und damit ein effektiveres Wissensmanagement ermöglichen, was den Wissenstransfer und die Einarbeitung von Personal erleichtert. Eine KI-gestützte Suchfunktion könnte relevante Methoden aus früheren Projekten vorschlagen. Auch in der Datenerhebung wäre sie hilfreich, indem sie Rohdaten auf Anomalien, fehlende Werte und inkonsistente Messreihen prüft.

Auch die Entwicklung einer Zertifizierungspraxis für Forschungslabore wäre denkbar, wie sie in anderen Bereichen bereits etabliert ist. Die Forschungsleitenden der Labore A bis E wurden bereits befragt, ob sie ein solches Vorgehen auch für Forschungslabore befürworten würden. Die Antworten fielen gemischt aus: Der Ansatz wurde nicht grundsätzlich abgelehnt, jedoch müssten verschiedene Aspekte berücksichtigt werden. Dazu zählt u. a., dass sich Labore durch ein solches Zertifikat nicht von ihrer eigenen Verantwortung entbinden dürfen. Der Aufwand muss – insbesondere mit Blick auf kleinere Forschungslabore – vertretbar bleiben, die Anforderungen sollten skalierbar sein und sowohl das Verfahren als auch die ausgestellten Zertifikate müssten vor Betrug und Fälschung geschützt werden. Für fundierte Überlegungen hinsichtlich einer Zertifizierung in der Forschung wäre künftig eine vertiefte Betrachtung von verschiedenen Forschungseinrichtungen, Interessenvertretungen wie der DFG, weiteren Fördermittelgebern sowie der forschenden Industrie notwendig. Zudem sollte eine Kosten-Nutzen-Analyse durchgeführt werden. Erste Ansätze hierzu sind bereits vorhanden [15, 16]. Der Mehrwert eines gemeinsamen Qualitätsstandards ist aus der Perspektive der Forschenden jedenfalls gegeben [17, 18].

Das Projekt zeigte, dass QM in der Forschung einen Mehrwert bieten kann und auch weiterempfohlen wird. Die Befragungen liefern jedoch lediglich erste Hinweise zur Akzeptanz und zum Nutzen von QM in der Forschung, sie erlauben keine generalisierbaren Aussagen. Die Ergebnisse stehen im Einklang mit früheren Studien, die ähnliche Vorteile eines QMS in anderen Forschungsumgebungen identifiziert haben [10–12], jedoch ist die Übertragbarkeit dieser Ergebnisse auf verschiedene Labortypen und -kontexte noch unklar. Da bislang keine vergleichbaren Studien vorliegen, ist ein Abgleich mit anderen Ergebnissen zur Akzeptanz eines QMS in der Forschung nicht möglich. Es wäre sinnvoll, die Ergebnisse dieser Studie mit bestehenden Theorien und Modellen des Qualitätsmanagements z. B. in weiteren wissenschaftlichen Abschlussarbeiten zu vergleichen, um die Relevanz und Anwendbarkeit der gewonnenen Erkenntnisse zu stärken. Die Auswahl der Pilotlabore sowie die Einbindung des RKI als Ressortforschungseinrichtung schränken die Aussagekraft des Projekts zusätzlich ein, da keine Repräsentativität für die wissenschaftliche Gemeinschaft besteht.

Der langfristige Nutzen des QMS in Bezug auf objektive Laborparameter, darunter Publikationsleistungen, Förderbewilligungen und -verlängerungen, Personalbindung und Qualifikationsabschlüsse des wissenschaftlichen Nachwuchses sowie die Reproduzierbarkeit der Forschung, und damit auf die nachhaltige Verwendung öffentlicher Mittel ist erst auf Basis weiterführender Datenerhebungen möglich. Dies setzt voraus, dass das System dauerhaft in den Arbeitsalltag integriert ist und erste Forschungsergebnisse unter seiner Anwendung veröffentlicht wurden. Erste Produktivitätssteigerungen wurden in den Befragungen bereits indirekt festgestellt und lassen sich auf ein im QMS verbessertes Ablagesystem, weniger Wiederholungen von Experimenten aufgrund von Fehlern sowie eine optimierte Kommunikation zurückführen (Abb. 2b–d). Zusätzlich wäre es wertvoll, die Erfahrungen anderer Forschungseinrichtungen zu dokumentieren, um ein umfassenderes Bild der Implementierung und Akzeptanz von QMS in der Forschung zu erhalten. Aus diesem Grund wäre es zielführend, die Erhebung in den Pilotlaboren zu einem späteren Zeitpunkt zu wiederholen und weitere Studien in Forschungslaboren unterschiedlicher Art und mit längeren Evaluationszeiträumen durchzuführen. Nur so lässt sich die Wirksamkeit des QMS umfassend bewerten und validieren.

Auch kann zukünftig die Studie um Untersuchungen in Industrieforschungslaboren erweitert werden. In diesen ist ein QMS häufig verpflichtend und Aspekte wie Dokumentation, Nachvollziehbarkeit sowie Risikomanagement sind fest verankert. Im Gegensatz dazu fehlen in der akademischen Forschung bislang weitgehend Anreize zur Implementierung von Qualitätsmanagement. Förderinstitutionen, wissenschaftliche Einrichtungen und Fachzeitschriften sollten daher gezielt Maßnahmen ergreifen, um Forschungslabore zu motivieren, ihre bestehenden Arbeitsabläufe kritisch zu reflektieren und mithilfe von QM-Strukturen systematisch zu optimieren. Solche Anreize könnten insbesondere darin bestehen, Qualität statt Quantität zu honorieren: Der Fokus sollte weniger auf der Anzahl von Publikationen und bibliometrischen Kennzahlen liegen, sondern stärker auf methodischer Sorgfalt, Replizierbarkeit und den Prinzipien von *Open Science*. *Open-Science*-Praktiken sollten in der Bewertung von Publikationen und Forschungsleistungen ein höheres Gewicht erhalten. Ebenso sollten Replikationsstudien verstärkt gefördert werden, beispielsweise im Rahmen spezifischer Förderprogramme oder Forschungspreise. Darüber hinaus sollte die wissenschaftliche Leistung von Forschenden an den jeweiligen Einrichtungen umfassender bewertet werden, etwa im Hinblick auf die methodische Qualität, die Durchführung von Replikationen und Open-Science-Beiträgen, die Förderung des wissenschaftlichen Nachwuchses, den Beitrag zur Forschungsintegrität, die Beteiligung an Peer-Review-Verfahren, Gremienarbeit sowie Engagement in der Lehre.

## Fazit

Jedes Labor hat individuelle Verbesserungsbedarfe. Ein starres, verpflichtendes QMS mit festgelegten Maßnahmen und Dokumentationsvorgaben ist für Forschungslabore daher wenig vorteilhaft. Vielmehr sollten QM-Maßnahmen gezielt an den Stellen ansetzen, an denen sie die Effizienz steigern, und in Zusammenarbeit mit dem Forschungsteam erarbeitet werden. Entscheidend sind dabei das Engagement von Mitarbeitenden und Leitung sowie eine ausgewogene Kosten-Nutzen-Relation, da Überregulierung kontraproduktiv wirken kann.

## Supplementary Information

ESM1: Zusatzmaterial 1

ESM2: Zusatzmaterial 2

ESM3: Zusatzmaterial 3

## Data Availability

Die zugrunde liegenden Rohdaten können bei dem Qualitätsmanagement des RKI unter qm@rki.de angefragt werden.
