# Supplementary material for: Quality management in research—the perspective of research teams
Source: Bundesgesundheitsblatt Gesundheitsforschung Gesundheitsschutz. 2026 Feb 10;69(3):347–59. [Article in German] doi: 10.1007/s00103-026-04191-0 (PMC12957627; doi:10.1007/s00103-026-04191-0)
Supplement: Supplementary file 1 — ESM1: Zusatzmaterial 1 [file 103_2026_4191_MOESM1_ESM.pdf]

QM in der Forschung – KAP-Befragung in biomedizinischen Forschungslaboren

Dieser Fragebogen widmet sich dem Erfassen von Wissen, Praxiserfahrungen und Einstellungen zu übergreifenden Themen von Forschungsprozessen und Qualitätsmanagement.

WISSEN ZU QUALITÄT UND QUALITÄTSMANAGEMENTMAßNAHMEN

In dem folgenden Fragen möchten wir mehr über Ihre Kenntnisse zu Qualitätsmanagementmaßnahmen erfahren. Bitte antworten Sie mit Ja oder Nein.

Frage Nr.	Fragestellung	Antwortoptionen	
		Ja	Nein
1	Ich weiß, wo die Anleitung für den Gebrauch und den Betrieb von Geräten und die Arbeitsanweisungen liegen. *(QMM1)*		
2	Ich weiß, wo ich nachsehen kann, wer in unserem Forschungsteam für welche Tätigkeit verantwortlich und geschult ist. *(QMM2)*		
3	Ich weiß, wie ich mich im Falle eines Verdachts von wissenschaftlichem Fehlverhalten verhalten soll? *(QMM3)*		
4	Ich weiß, dass die mangelnde Reproduzierbarkeit von Forschungsergebnissen in der Wissenschaftsgemeinschaft (Replication Crisis) zunehmend ein Problem darstellt. *(QMM4)*		

INDIVIDUELLE QUALITÄTSGESICHERTE FORSCHUNG

Im Folgenden finden Sie einige Aussagen zur qualitätsgesicherten Forschung. Bitte geben Sie an, inwiefern diese Aussagen auf ihre Praxis im Labor zutreffen, so wie sie diese im letzten Jahr ausgeübt haben.

Frage Nr.	Fragestellung	Antwortoptionen				
		**trifft nicht zu** (1)	**trifft eher nicht zu** (2)	**Unent-schieden** (3)	**trifft eher zu** (4)	**trifft zu** (5)
1	Ich dokumentiere meine Daten umgehend, sobald ich sie generiert habe. *(QS1)*					
2	Ich notiere mir die Ergebnisse eines Versuches erst handschriftlich, bevor ich sie in den Computer übertrage. *(QS2)*					

Frage Nr.	Fragestellung	Antwortoptionen				
		**trifft nicht zu** (1)	**trifft eher nicht zu** (2)	**Unent-schieden** (3)	**trifft eher zu** (4)	**trifft zu** (5)
3	Für die Dokumentation des Forschungsprozesses nutze ich Vorlagen/Templates. *(QS3)*					
4	Wenn ich im Forschungsbereich von einer standardisierten Arbeitsanweisung (SOP) abweiche, notiere ich dies nachvollziehbar. *(QS4)*					
5	Ich mache mir vor der Durchführung des Versuchs Gedanken zur statistischen Auswertung (z.B. Stichprobenahme, Fallzahlen). *(QS5)*					
6	Ich komme in unserem Ablagesystem schnell an die Dokumente bzw. Daten heran, die ich benötige. *(QS6)*					

Im Folgenden finden Sie einige Aussagen zur qualitätsgesicherten Forschung. Bitte geben Sie an, wie häufig die unten genannten Ereignisse in Ihrer alltäglichen Forschungspraxis im letzten Jahr vorgekommen sind.

Frage Nr.	Fragestellung	Antwortoptionen				
		**nie** (1)	**selten** (2)	**Gelegent-lich** (3)	**häufig** (4)	**sehr häufig** (5)
7	Verwenden Sie Materialien über das Mindesthaltbarkeitsdatum hinaus ohne Prüfung auf Verwendbarkeit (z.B. durch Bestimmung von Identität (z.B. bei Zelllinien) oder Reinheit?)? *(QS7)*					
8	Wie häufig müssen Sie Arbeiten wiederholen, weil Mitarbeitende krank werden oder ausscheiden und ihr Wissen/ihre Arbeit nicht übergeben haben? *(QS8)*					
9	Wie häufig kommt es vor, dass Sie nicht wissen, was der Kollege oder die Kollegin vor Ihnen gemacht hat, bzw. auf welchem Stand Sie sind. *(QS9)*					

QUALITÄT IM TEAM

Im Folgenden geht es um die qualitätsgesicherte Forschungspraxis in Ihrem Labor bzw. Forschungsteam.

Bitte geben Sie an, inwiefern diese Aussagen aus Ihrer Sicht auf die gängige Praxis im Labor, so wie sie diese im letzten Jahr erlebt haben, zutreffen.

Frage Nr.	Fragestellung	Antwortoptionen					
		**nie** (1)	**selten** (2)	**Gelegent -lich** (3)	**häufig** (4)	**sehr häufig** (5)	**Weiß nicht** (9)
1	Für Publikationen werden validierte/verifizierte Methoden benutzt. *(QT1)*						
2	Mitarbeitende werden für die Geräte eingearbeitet, die sie in ihrem Tätigkeitsbereich nutzen (zum Beispiel über Arbeitsanweisungen oder persönliches Mentoring). *(QT2)*						
3	Geräte, die nicht funktionieren, werden außer Betrieb genommen und sichtbar als defekt gekennzeichnet. *(QT3)*						
4	Es gibt in unserem Team Absprachen, welche Fehler zu dokumentieren sind. *(QT4)*						
5	Wenn ein Fehler gemeldet wird, wird die Fehlerursache ermittelt. *(QT5)*						
6	Würden Sie sagen, dass Sie (und Ihr Team) zeitlich unter Druck gesetzt waren, die Ergebnisse schnell zu erzielen, auszuwerten und zu veröffentlichen (z.B. durch Förderperioden)? *(QT6)*						
7	Neue Mitarbeitende werden in unserem Laborteam von einer Mentorin oder einem Mentor begleitet. *(QT7)*						
8	Es findet ein regelmäßiger Austausch in Form eines Meetings im Forschungsteam statt. *(QT8)*						
9	In unserem Team kann jeder seine bzw. ihre Ideen und Vorschläge offen ansprechen. *(QT9)*						
10	Wie häufig kommt es vor, dass in Ihrer Arbeitsgruppe Experimente/Versuche aufgrund von Fehlern wiederholt werden müssen (z.B. durch lückenhafte Dokumentation, falsches Pipettieren, falsche Bedienung des Gerätes, etc.)? *(QT10)*						

Wir möchten mehr über die Situation in Ihrem Labor erfahren. Bitte antworten Sie mit ja oder nein.

Frage Nr.	Fragestellung	Antwortoptionen		
		Ja	Nein	Weiß nicht
11	Ist in ihrem Labor der Kalibrierstatus eines jeden Gerätes klar ersichtlich? *(QT11)*			
12	Gibt es eine aktuelle Liste zum Wartungsstatus eines jeden Gerätes im Labor? *(QT12)*			
13	Gibt es einen oder mehrere Geräteverantwortlichen für die Geräte, die in Ihrem Labor genutzt werden? *(QT13)*			
14	Existiert ein Einarbeitungsplan für neue Mitarbeiter, Auszubildende, Studierende, Praktikanten? *(QT14)*			
15	Die Teamsitzungen werden protokolliert. *(QT15)*			

Wir würde gerne mehr über die Art und Weise, wie sie dokumentieren, erfahren und wo sie Unterstützung brauchen.

Frage Nr.	Fragestellung	Antwortoptionen
16	Welche Hilfsmittel für die Dokumentation der Forschungsergebnisse gibt es in Ihrem Labor/Team (z.B. Laborbuch, LIMS, Formblätter zum Ausfüllen, Exceltabellen)? *(QT16)*	
17	Was kann Ihrer Meinung nach in Ihrem Labor/in Ihrem Team bezüglich Qualitätssicherung noch verbessert werden (Bsp. mehr Schulungsmöglichkeiten)? *(QT17)*	
18	Für die Dokumentation der Daten wünsche ich mir… (Mehrfachantworten möglich) *(QT18)*	○ Richtlinien<br>○ ein elektronisches Laborbuch<br>○ Vorgabeblätter/Templates<br>○ ein Labor Information Management System (LIMS)<br>○ Checklisten<br>○ eine zweite Person zur Unterstützung<br>○ Offen:

QUALITÄT IM FORSCHUNGSPROZESS

ALLGEMEIN

Im Folgenden finden Sie einige Aussagen zur qualitätsgesicherten Forschung. Bitte geben Sie an, wie weit Sie den folgenden Aussagen zustimmen bzw. nicht zustimmen.

Frage Nr.	Fragestellung	Antwortoptionen					
		stimme überhaupt nicht zu (1)	stimme nicht zu (2)	teils teils (3)	stimme zu (4)	stimme voll zu (5)	weiß nicht (9)
1	Mir ist es wichtig, Reagenzien, die ich länger nutze als vorgeschrieben, zu kontrollieren, ob diese noch verwendbar sind. *(Q1)*						
2	Ich finde es gut, wenn die Nutzung von gemeinschaftlich genutzten Geräten über Nutzerlisten dokumentiert wird. *(Q2)*						
3	Die Ordnerstruktur sollte so aufgebaut sein, so dass auch neue Mitarbeiter sich leicht zurechtfinden. *(Q3)*						
4	Arbeits- und Geräteanweisungen in unserem Labor sind für mich unverständlich formuliert. *(Q4)*						
5	Es wäre gut, wenn auch nicht gelungene Versuche publiziert oder in Open Access Datenbanken zur Verfügung gestellt werden würden. *(Q5)*						

QUALITÄTSMANAGEMENT

Im Folgenden finden Sie nun einige Aussagen zu Qualitätsinstrumenten und Qualitätsmanagement in der Forschung im Allgemeinen. Bitte geben Sie an, wie weit Sie den folgenden Aussagen zustimmen bzw. nicht zustimmen.

Frage Nr.	Fragestellung	Antwortoptionen					
		stimme überhaupt nicht zu (1)	stimme nicht zu (2)	teils teils (3)	stimme zu (4)	stimme voll zu (5)	weiß nicht (9)
1	Nachvollziehbarkeit und Wiederholbarkeit sind wichtige Qualitätsmerkmale der Forschung. *(QM1)*						
2	Drittmittelanträge (bzw. Publikation) würden von einem objektiven Qualitätsnachweis profitieren. *(QM2)*						
3	Ein Qualitätsmanagementerleichtert es, Daten für Anschlussprojekte zu nutzen. *(QM3)*						
4	Kreativität in der Forschung und Qualitätsmanagement schließen sich nicht aus. *(QM4)*						
5	Qualitätsmanagement erfordert zu viel Zeit im Vergleich zum Nutzen. *(QM5)*						

Im Folgenden wollen wir Ihnen einige Aussagen vorstellen, die darauf zielen, wie Qualitätsmanagementinstrumente die Qualität in der Forschung unterstützen können. Bitte geben Sie an, inwieweit sie den folgenden Aussagen zustimmen.

Frage Nr.	Fragestellung	Antwortoptionen					
		stimme überhaupt nicht zu (1)	stimme nicht zu (2)	teils teils (3)	stimme zu (4)	stimme voll zu (5)	weiß nicht (9)
6	Ich wünsche mir ein anonymes Fehlermeldesystem. *(QM6)*						
7	Ich wünsche mir eine bessere Betreuung des wissenschaftlichen Nachwuchses. *(QM7)*						
8	Ich wünsche mir… *(QM8)*	____________					

Zum Schluss möchten wir Ihnen noch gerne einige Fragen zu Qualitätsmanagement in der Forschung im Allgemeinen stellen.

Frage Nr.	Fragestellung	Antwortoptionen
9	Ich habe eine positive Einstellung gegenüber Qualitätsmanagement, weil es …… *(QM9)*	○ … sich positiv auf meine Arbeit auswirken kann.<br>○ … die benötigte Arbeitszeit durch vereinfachte Abläufe verkürzen kann.
10	Ich sehe folgende Chancen in Qualitätsmanagementmaßnahmen in der Forschung: *(QM10)*	______________________
11	Ich habe folgende Bedenken gegenüber Qualitätsmanagementmaßnahmen in der Forschung: *(QM11)*	______________________

Bitte geben Sie an, wie weit Sie den folgenden Aussagen zustimmen bzw. nicht zustimmen.

Frage Nr.	Fragestellung	Antwortoptionen		
		Ja	Nein	Weiß nicht
1	Qualitätsmanagement hilft dabei, meine Leistung bei der Institutsleitung geltend zu machen. *(LTQM1)*			
2	Sind Sie der Meinung, dass gemeinsame Qualitätsstandards in der Forschung einen positiven Impact auf die biomedizinische Forschung haben könnten? *(LTQM2)*			

Frage Nr.	Fragestellung	Antwortoptionen		
		Ja	Nein	Weiß nicht
3	Wenn ja, warum? *(LTQM3)*	○ Um sich auf eine gemeinsame Qualitätsbasis zu verständigen<br>○ Erfahrungswissen auszutauschen<br>○ Um Vergleichbarkeit zu schaffen<br>○ Um eine Reproduzierbarkeit der Experimente zu gewährleisten<br>○ Um die Effizienz der Arbeitsabläufe zu steigern<br>○ Offen:		
4	Was kann der Mehrwehrt von internen Audits sein? *(LTQM4)*	○ fachlicher Austausch<br>○ Effizienzsteigerung der Prozesse und Abläufe<br>○ Fehleranalyse<br>○ Verbesserte Einarbeitung neuer Mitarbeiter<br>○ Überprüfung der Arbeitsauslastung der Mitarbeiter<br>○ Offen:		

REPLIZIERBARKEIT IN DER FORSCHUNG

Wir möchten nun spezifischer zur Replizierbarkeit von Versuchen fragen. Da es keine einheitliche Definition des Begriffs „Replizierbarkeit“ gibt, finden Sie vorher die Definition der DFG. So sprechen wir vom selben Thema:

Info: Begrifflichkeiten Replizierbarkeit und Reproduzierbarkeit *(DFG, https://www.dfg.de/download/pdf/dfg_im_profil/geschaeftsstelle/publikationen/stellungnahmen_papiere/2018/180507_stellungnahme_replizierbarkeit_sgkf.pdf, 22.06.2023):*

„Die Begriffsbildung der Wörter Replikation, Replizierbarkeit bzw. der ebenfalls oft verwendeten Begriffe Reproduktion, Reproduzierbarkeit sowie die Abgrenzungen der Bedeutung zwischen diesen Ausdrücken ist noch nicht abgeschlossen. Die Begriffe werden auch im angelsächsischen Sprachraum nicht einheitlich verwendet.

Replikation ist die Wiederholung einer Untersuchung/eines Experiments/einer Studie, die den Anspruch auf Wiederholbarkeit erhebt, meint aber auch allgemein die ergebnisoffene Möglichkeit, etwas zu wiederholen bzw. ein Experiment noch einmal durchführen zu können. Replizierbarkeit meint entsprechend die Möglichkeit bzw. Fähigkeit, Ergebnisse innerhalb des Fehlerrahmens wiederholend zu bestätigen.“

Frage Nr.	Fragestellung	Antwortoptionen		
		**Ja**	**Nein**	**Weiß nicht**
1	Kommt es vor, dass Sie eigene oder fremde Experimente replizieren? *(LTQS1)*			
2	Wenn Ja: Gelingt dies immer? *(LTQS2)*			
3	Wenn Nein: Was sind die Gründe? Mehrfachantworten sind möglich. *(LTQS3)*	○ Die Veröffentlichung hat statistische Mängel<br>○ Fehlerhafte Methodenangabe<br>○ Sonstige:<br>____________________		

Frage Nr.	Fragestellung	Antwortoptionen		
		Ja	Nein	Weiß nicht
4	Welche Faktoren führen Ihrer Meinung nach zu einer geringen Replizierbarkeit in der biomedizinischen Forschung? *(LTQS4)*			
5	Welche Faktoren könnten die Replizierbarkeit in der biomedizinischen Forschung verbessern? *(LTQS5)*			

Vielen Dank, dass Sie an unserer Befragung teilgenommen haben! Wir wissen Ihre Zeit und Mühe zu schätzen! Sollten Sie weitere Anmerkungen oder Fragen haben, zögern Sie bitte nicht, uns zu kontaktieren.

Haben Sie noch weitere Anmerkungen/Hinweise/Feedback für uns?
