# Supplementary material for: Quality management in research—the perspective of research teams
Source: Bundesgesundheitsblatt Gesundheitsforschung Gesundheitsschutz. 2026 Feb 10;69(3):347–59. [Article in German] doi: 10.1007/s00103-026-04191-0 (PMC12957627; doi:10.1007/s00103-026-04191-0)
Supplement: Supplementary file 2 — ESM2: Zusatzmaterial 2 [file 103_2026_4191_MOESM2_ESM.pdf]

QM in der Forschung – KAP-Befragung in Forschungslaboren

Dieser Fragebogen widmet sich dem Erfassen von Wissen, Praxiserfahrungen und Einstellungen zu übergreifenden Themen von Forschungsprozessen und Qualitätsmanagement.

WISSEN ZU QUALITÄT UND QUALITÄTSMANAGEMENTMAẞNAHMEN

In dem folgenden Fragen möchten wir mehr über Ihre Kenntnisse zu Qualitätsmanagementmaßnahmen erfahren. Bitte antworten Sie mit Ja oder Nein.

Frage Nr.	Fragestellung	Antwortoptionen	
		Ja	Nein
1	Ich weiß, wo die Anleitung für den Gebrauch und den Betrieb von Geräten und die Arbeitsanweisungen liegen. *(QMM1)*		
2	Ich weiß, wer in unserem Team für was verantwortlich ist. *(Neu 1)*		
3	Ich weiß, wo ich nachsehen kann, wer in unserem Forschungsteam für welche Tätigkeit verantwortlich und geschult ist. *(QMM2)*		
4	Ich weiß, wie ich mich im Falle eines Verdachts von wissenschaftlichem Fehlverhalten verhalten soll? *(QMM3)*		

IHRE ALLGEMEINE EINSTELLUNG

Frage Nr.	Fragestellung	Antwortoptionen				
		trifft nicht zu (1)	trifft eher nicht zu (2)	Unent-schieden (3)	trifft eher zu (4)	trifft zu (5)
1	Ich fühle mich in der Guten Wissenschaftlichen Praxis (GWP) sicher. *(Neu 2)*					
2	Ich finde mich in den Laborbüchern meiner Kollegen zurecht. *(Neu 3)*					
3	Mir würde eine Übersicht, wer in was eingearbeitet ist, helfen. *(Neu 4)*					

INDIVIDUELLE QUALITÄTSGESICHERTE FORSCHUNG

Im Folgenden finden Sie einige Aussagen zur qualitätsgesicherten Forschung. Bitte geben Sie an, inwiefern diese Aussagen auf ihre Praxis im Labor zutreffen, so wie sie diese seit der letzten Befragung (Januar 2024) ausgeübt haben.

Frage Nr.	Fragestellung	Antwortoptionen				
		**trifft nicht zu** (1)	**trifft eher nicht zu** (2)	**Unent-schieden** (3)	**trifft eher zu** (4)	**trifft zu** (5)
1	Ich dokumentiere meine Daten umgehend, sobald ich sie generiert habe. *(QS1)*					
2	Ich notiere mir die Ergebnisse eines Versuches erst handschriftlich, bevor ich sie in den Computer übertrage. *(QS2)*					
3	Für die Dokumentation des Forschungsprozesses nutze ich Vorlagen/Templates. *(QS3)*					
4	Wenn ich im Forschungsbereich von einer standardisierten Arbeitsanweisung (SOP) abweiche, notiere ich dies nachvollziehbar. *(QS4)*					
5	Ich mache mir vor der Durchführung des Versuchs Gedanken zur statistischen Auswertung (z.B. Stichprobenahme, Fallzahlen). *(QS5)*					
6	Ich komme in unserem Ablagesystem schnell an die Dokumente bzw. Daten heran, die ich benötige. *(QS6)*					

Im Folgenden finden Sie einige Aussagen zur qualitätsgesicherten Forschung. Bitte geben Sie an, wie häufig die unten genannten Ereignisse in Ihrer alltäglichen Forschungspraxis seit der letzten Befragung (Januar 2024) vorgekommen sind.

Frage Nr.	Fragestellung	Antwortoptionen				
		**nie** (1)	**selten** (2)	**Gelegent-lich** (3)	**häufig** (4)	**sehr häufig** (5)
7	Verwenden Sie Materialien über das Mindesthaltbarkeitsdatum hinaus ohne Prüfung auf Verwendbarkeit (z.B. durch Bestimmung von Identität (z.B. bei Zelllinien) oder Reinheit?)? *(QS7)*					
8	Wie häufig müssen Sie Arbeiten wiederholen, weil Mitarbeitende krank werden oder ausscheiden und ihr Wissen/ihre Arbeit nicht übergeben haben? *(QS8)*					

Frage Nr.	Fragestellung	Antwortoptionen				
		**nie** (1)	**selten** (2)	**Gelegent -lich** (3)	**häufig** (4)	**sehr häufig** (5)
9	Wie häufig kommt es vor, dass Sie nicht wissen, was der Kollege oder die Kollegin vor Ihnen gemacht hat, bzw. auf welchem Stand Sie sind. *(QS9)*					

QUALITÄT IM TEAM

Im Folgenden geht es um die qualitätsgesicherte Forschungspraxis in Ihrem Labor bzw. Forschungsteam.

Bitte geben Sie an, inwiefern diese Aussagen aus Ihrer Sicht auf die gängige Praxis im Labor, so wie sie diese seit der letzten Befragung (Januar 2024) erlebt haben, zutreffen.

Frage Nr.	Fragestellung	Antwortoptionen					
		**nie** (1)	**selten** (2)	**Gele-gentlich** (3)	**häufig** (4)	**sehr häufig** (5)	**Weiß nicht** (9)
1	Für Publikationen werden validierte/verifizierte Methoden benutzt. *(QT1)*						
2	Mitarbeitende werden für die Geräte eingearbeitet, die sie in ihrem Tätigkeitsbereich nutzen (zum Beispiel über Arbeitsanweisungen oder persönliches Mentoring). *(QT2)*						
3	Geräte, die nicht funktionieren, werden außer Betrieb genommen und sichtbar als defekt gekennzeichnet. *(QT3)*						
4	Es gibt in unserem Team Absprachen, welche Fehler zu dokumentieren sind. *(QT4)*						
5	Wenn ein Fehler gemeldet wird, wird die Fehlerursache ermittelt. *(QT5)*						
6	Neue Mitarbeitende werden in unserem Team von einer Mentorin oder einem Mentor begleitet. *(QT7)*						
7	Es findet ein regelmäßiger Austausch in Form eines Meetings im Forschungsteam statt. *(QT8)*						

Frage Nr.	Fragestellung	Antwortoptionen					
		**nie** (1)	**selten** (2)	**Gele-gentlich** (3)	**häufig** (4)	**sehr häufig** (5)	**Weiß nicht** (9)
8	In unserem Team kann jeder seine bzw. ihre Ideen und Vorschläge offen ansprechen. *(QT9)*						
9	Wie häufig kommt es vor, dass in Ihrer Arbeitsgruppe Experimente/Versuche aufgrund von Fehlern wiederholt werden müssen (z.B. durch lückenhafte Dokumentation, falsches Pipettieren, falsche Bedienung des Gerätes, etc.)? *(QT10)*						

Wir möchten mehr über die Situation in Ihrem Labor erfahren. Bitte antworten Sie mit ja oder nein.

Frage Nr.	Fragestellung	Antwortoptionen		
		**Ja**	**Nein**	**Weiß nicht**
10	Ist in ihrem Labor der Kalibrierstatus eines jeden Gerätes klar ersichtlich? *(QT11)*			
11	Gibt es eine aktuelle Liste zum Wartungsstatus eines jeden relevanten Gerätes im Labor? *(QT12)*			
12	Gibt es einen oder mehrere Geräteverantwortlichen für die Geräte, die in Ihrem Labor genutzt werden? *(QT13)*			
13	Existiert ein Einarbeitungsplan für neue Mitarbeiter, Auszubildende, Studierende, Praktikanten? *(QT14)*			
14	Die Teamsitzungen werden protokolliert. *(QT15)*			

QUALIITÄT IM FORSCHUNGSPROZESS

ALLGEMEIN

Im Folgenden finden Sie einige Aussagen zur qualitätsgesicherten Forschung. Bitte geben Sie an, wie weit Sie den folgenden Aussagen zustimmen bzw. nicht zustimmen.

Frage Nr.	Fragestellung	Antwortoptionen					
		**stimme überhaupt nicht zu** (1)	**stimme nicht zu** (2)	**teils teils** (3)	**stimme zu** (4)	**stimme voll zu** (5)	**weiß nicht** (9)
1	Mir ist es wichtig, Reagenzien, die ich länger nutze als vorgeschrieben, zu kontrollieren, ob diese noch verwendbar sind. *(Q1)*						
2	Ich finde es gut, wenn die Nutzung von gemeinschaftlich genutzten Geräten über Nutzerlisten dokumentiert wird. *(Q2)*						
3	Arbeits- und Geräteanweisungen in unserem Labor sind für mich unverständlich formuliert. *(Q4)*						

QUALITÄTSMANAGEMENT

Im Folgenden finden Sie nun einige Aussagen zu Qualitätsinstrumenten und Qualitätsmanagement in der Forschung im Allgemeinen. Bitte geben Sie an, wie weit Sie den folgenden Aussagen zustimmen bzw. nicht zustimmen.

Frage Nr.	Fragestellung	Antwortoptionen					
		**stimme überhaupt nicht zu** (1)	**stimme nicht zu** (2)	**teils teils** (3)	**stimme zu** (4)	**stimme voll zu** (5)	**weiß nicht** (9)
1	Drittmittelanträge (bzw. Publikation) würden von einem objektiven Qualitätsnachweis profitieren. *(QM2)*						
2	Ein Qualitätsmanagement erleichtert es, Daten für Anschlussprojekte zu nutzen. *(QM3)*						
3	Kreativität in der Forschung und Qualitätsmanagement schließen sich nicht aus. *(QM4)*						

Frage Nr.	Fragestellung	Antwortoptionen					
		**stimme überhaupt nicht zu** (1)	**stimme nicht zu** (2)	**teils teils** (3)	**stimme zu** (4)	**stimme voll zu** (5)	**weiß nicht** (9)
4	Qualitätsmanagement erfordert zu viel Zeit im Vergleich zum Nutzen. *(QM5)*						

Zum Schluss möchten wir Ihnen noch gerne einige Fragen zu Ihrem Qualitätsmanagement in der Forschung im Allgemeinen und im Vergleich zu Ihrer Arbeitspraxis vor der letzten Befragung (Januar 2024) stellen.

Frage Nr.	Fragestellung	Antwortoptionen
1	Was hat sich im Rahmen der eingeführten Qualitätsmanagementmaßnahmen in Ihrem Labor im Vergleich zu vorher verändert? *(Neu 5)*<br><br>(z.B. Umgang mit SOPs, Projektkommunikation, Validierung/Verifizierung, Wartung von Geräten, Klärung und Dokumentation von Verantwortlichkeiten, Feedbackmanagement, Dokumentation über selbst hergestellte Reagenzien (Herstellungsdatum, Haltbarkeit, etc.) Überarbeitung Ablagestruktur, Überwachung der Haltbarkeit von Reagenzien, Aufzeichnungen, systematische Einarbeitung/Verabschiedung, etc.)	
2	Was hat sich durch die Qualitätsmanagementmaßnahmen in Ihrem Forschungslabor/ im Team verbessert? *(Neu 6)*	
3	Was hat sich durch die Qualitätsmanagementmaßnahmen in Ihrem Forschungslabor/ im Team verschlechtert? *(Neu 7)*	

Frage Nr.	Fragestellung	Antwortoptionen
4	Haben Sie persönlich Vorteile oder Nachteile durch die Qualitätsmanagementmaßnahmen erlebt? *(Neu 8)*	○ Ja<br>○ Nein<br>○ Weiß nicht
4.1	Wenn ja, welche? *(Neu 9)*	____________________<br>____________________<br>____________________
5	Schränken die Qualitätsmanagementmaßnahmen Sie in Ihrer Kreativität ein? *(Neu 10)*	○ Ja<br>○ Nein<br>○ Weiß nicht
5.1	Wenn ja, wie? *(Neu 11)*	____________________<br>____________________<br>____________________<br>____________________
6	Ist nach Ihrer eigenen Erfahrung, die Sie mit dem System gemacht haben, dass Qualitätsmanagement zu stark von Vorurteilen vorbelastet? *(Neu 12)*	○ Ja<br>○ Nein<br>○ Weiß nicht
7	Hat sich Ihr Qualitätsverständnis verändert? *(Neu 13)*	○ Ja<br>○ Nein<br>○ Weiß nicht
7.1	Wenn ja, wie? *(Neu 14)*	____________________<br>____________________<br>____________________
8	Würden Sie ein Qualitätsmanagementsystem in der Forschung anderen Forschungsteams weiterempfehlen? *(Neu 15)*	○ Ja<br>○ Nein<br>○ Weiß nicht

Bitte geben Sie an, wie weit Sie den folgenden Aussagen zustimmen bzw. nicht zustimmen.

Frage Nr.	Fragestellung	Antwortoptionen		
		**Ja**	**Nein**	**Weiß nicht**
1	Sind Sie der Meinung, dass gemeinsame Qualitätsstandards in der Forschung einen positiven Impact auf die Forschung haben könnten? *(LTQM2)*			
2	Wenn ja, warum? *(LTQM3)*	o Um sich auf eine gemeinsame Qualitätsbasis zu verständigen<br>o Erfahrungswissen auszutauschen<br>o Um Vergleichbarkeit zu schaffen<br>o Um eine Reproduzierbarkeit der Experimente zu gewährleisten<br>o Um die Effizienz der Arbeitsabläufe zu steigern<br>o Offen: ____________		

Vielen Dank, dass Sie an unserer Befragung teilgenommen haben! Wir wissen Ihre Zeit und Mühe zu schätzen! Sollten Sie weitere Anmerkungen oder Fragen haben, zögern Sie bitte nicht, uns zu kontaktieren.

Haben Sie noch weitere Anmerkungen/Hinweise/Feedback für uns?

____

____

____

____

____

____
