# Supplementary material for: Quality management in research—the perspective of research teams
Source: Bundesgesundheitsblatt Gesundheitsforschung Gesundheitsschutz. 2026 Feb 10;69(3):347–59. [Article in German] doi: 10.1007/s00103-026-04191-0 (PMC12957627; doi:10.1007/s00103-026-04191-0)

Abbildung Z1 Vergleich KAP1 und KAP2 Pilotlabore

Ergebnisse der KAP1 und KAP2 Befragungen der Pilotlabore. Pilotlabore setzten unterschiedliche Schwerpunkte bei QMS-Einführung, sodass geringe oder fehlende Veränderungen nicht zwangsläufig eine Wirkung oder Wirkungslosigkeit widerspiegeln, sondern vielmehr, dass dieser Aspekt im jeweiligen Labor noch nicht bearbeitet wurde. Einige Verschlechterungen zwischen KAP1 und KAP2 weisen darauf hin, dass umso mehr Handlungsbedarf besteht.

Z1a Arbeits- und Geräteanweisungen in unserem Labor sind für mich unverständlich formuliert.

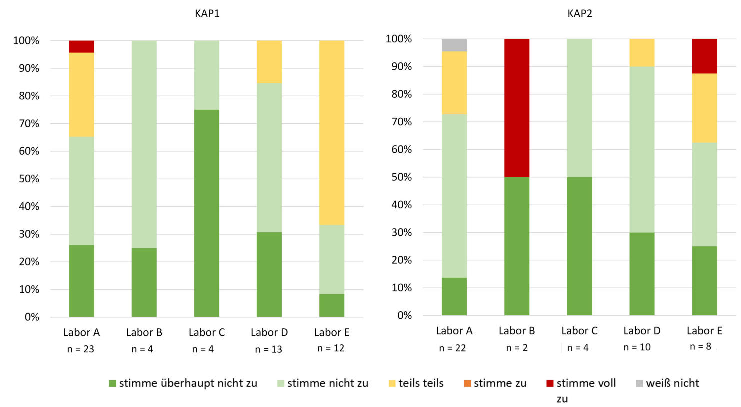

Z1e Gibt es eine aktuelle Liste zum Wartungsstatus eines jeden Gerätes im Labor?

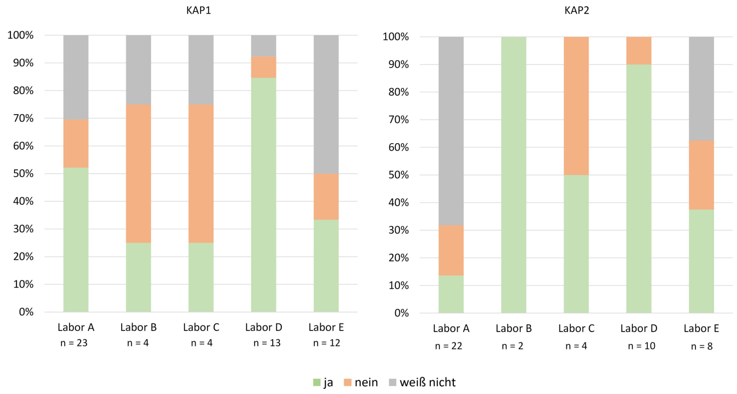

Z1b Gibt es einen oder mehrere Geräteverantwortlichen für die Geräte, die in Ihrem Labor genutzt werden?

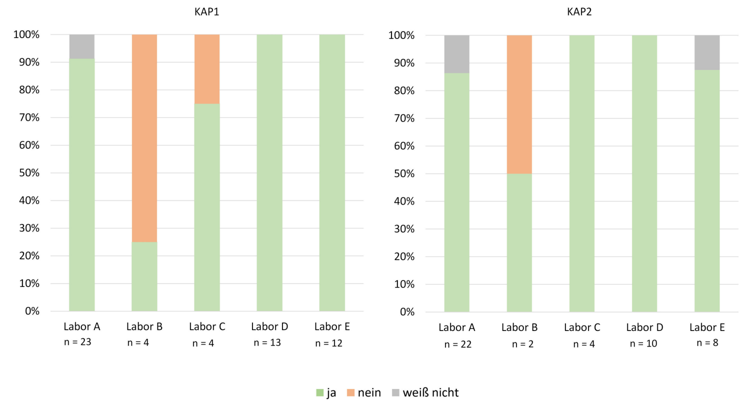

Z1f Ist in ihrem Labor der Kalibrierstatus eines jeden Gerätes klar ersichtlich?

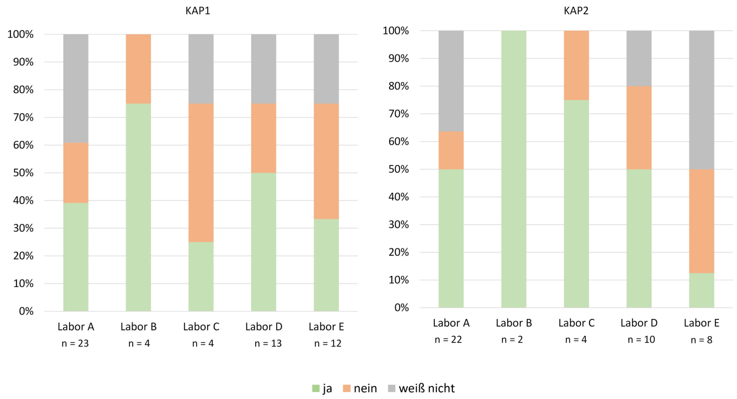

Z1c Die Teamsitzungen werden protokolliert.

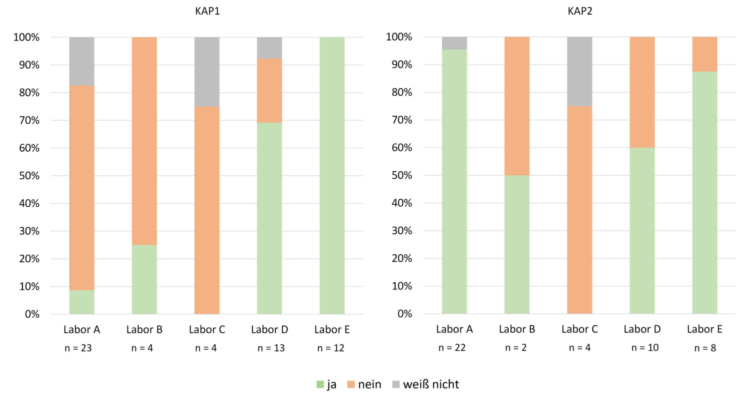

Z1g Ich komme in unserem Ablagesystem schnell an die Dokumente bzw. Daten heran, die ich benötige.

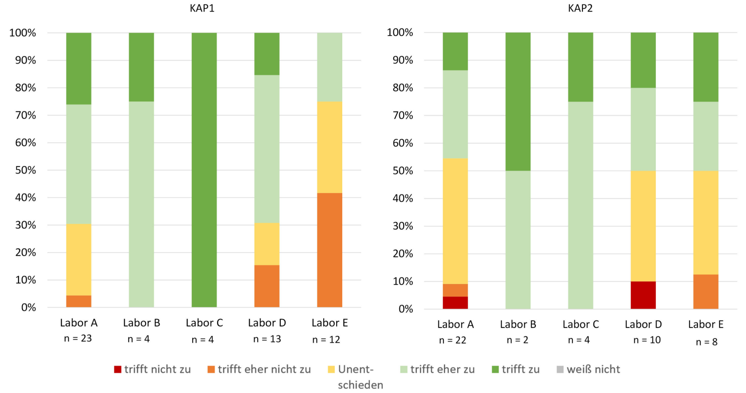

Z1d Existiert ein Einarbeitungsplan für neue Mitarbeiter, Auszubildende, Studierende, Praktikanten?

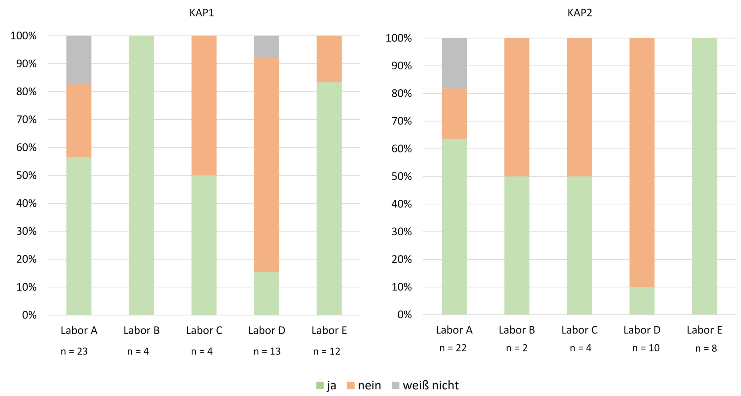

Abbildung Z2 Vergleich Organisation mit und ohne QM

Vergleich der KAP1 Pilotlabore mit V1 und V2. V1 besitzt seit über 10 Jahren ein QMS in Forschungslaboren. V2 löste nach 10 Jahren das Forschungs-QMS ungeordnet auf. V2 entspricht einem Labor ohne QM, jedoch mit verbliebenen QM-relevanten Kenntnissen. Rundungsbedingt können in den Grafiken geringfügige Abweichungen auftreten, wodurch die Ergebnisse nicht immer 100% ergeben.

Z2a Ich dokumentiere meine Daten umgehend, sobald ich sie generiert habe.

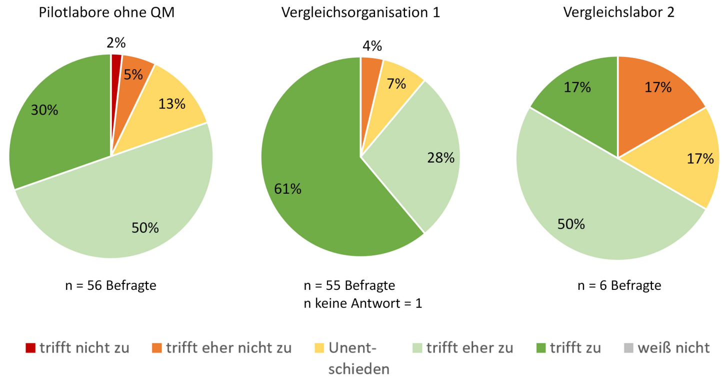

Z2e Verwenden Sie Materialien über das Mindesthaltbarkeitsdatum hinaus ohne Prüfung auf Verwendbarkeit (z.B. durch Bestimmung von Identität (z.B. bei Zelllinien) oder Reinheit)?

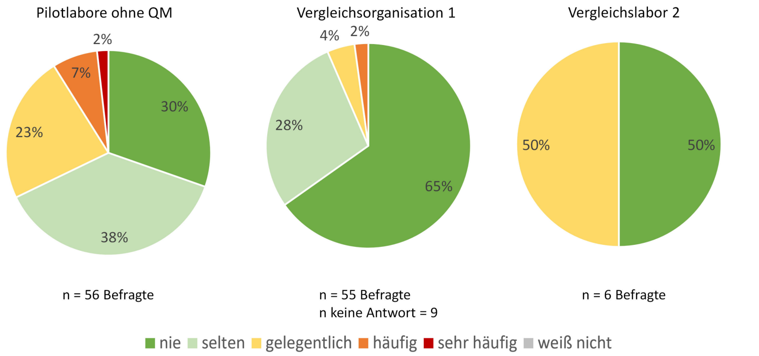

Z2b Die Teamsitzungen werden protokolliert.

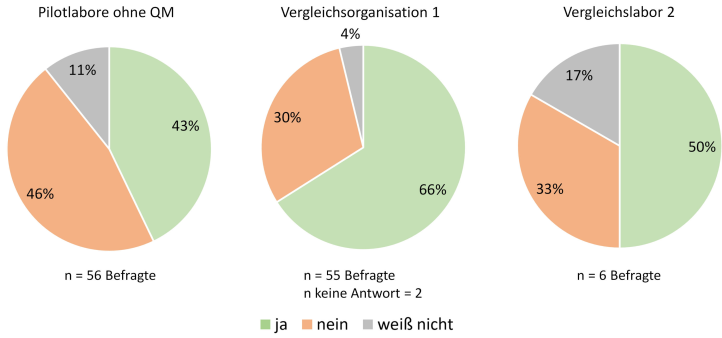

Z2f Mir ist es wichtig, Reagenzien, die ich länger nutze als vorgeschrieben, zu kontrollieren, ob diese noch verwendbar sind.

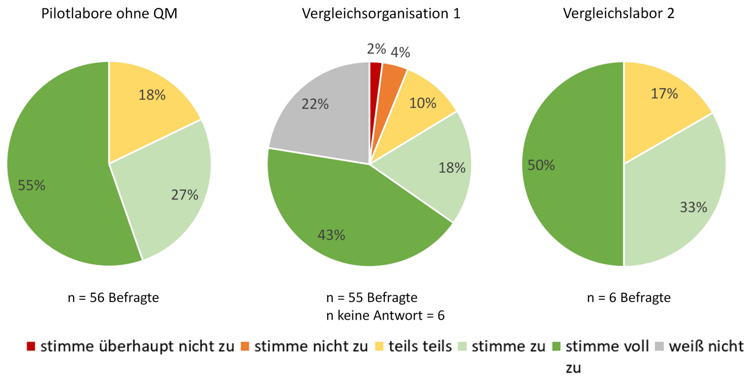

Z2c Existiert ein Einarbeitungsplan für neue Mitarbeiter, Auszubildende, Studierende, Praktikanten?

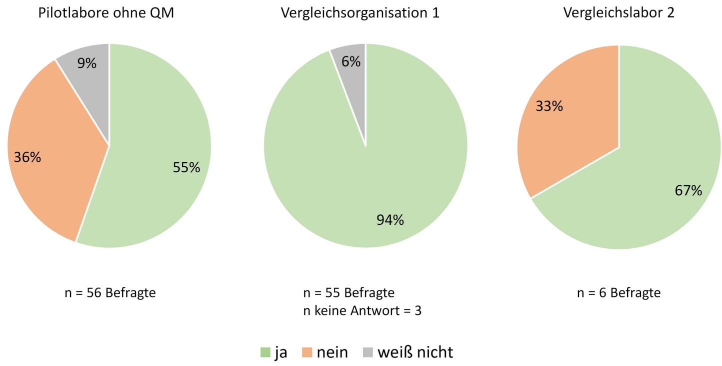

Z2g Geräte, die nicht funktionieren, werden außer Betrieb genommen und sichtbar als defekt gekennzeichnet.

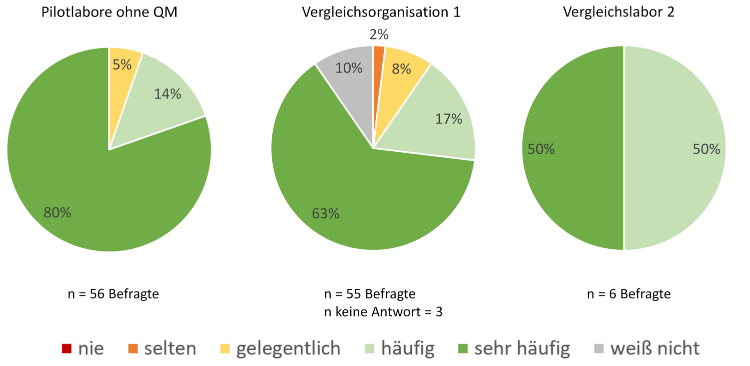

Z2d Wenn ich im Forschungsbereich von einer standardisierten Arbeitsanweisung (SOP) abweiche, notiere ich dies nachvollziehbar.

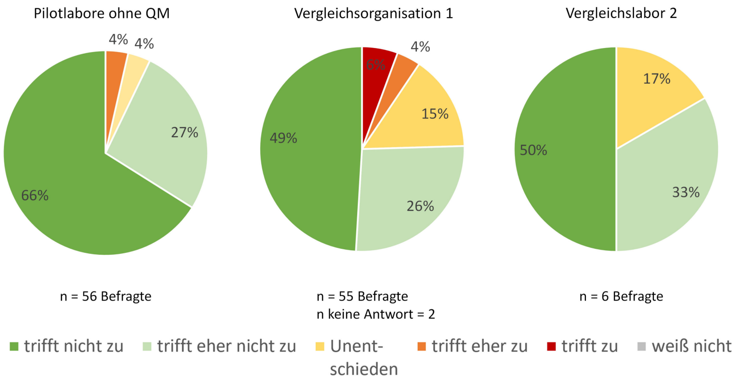

Abbildung Z3 Verbesserungen und Verschlechterungen durch ein QMS

Z3a genannte Verbesserungen von 83% der Mitarbeitenden der Pilotlabore; Z3b genannte Verbesserungen von 50% der Mitarbeitenden der V1; Z3c genannte Verschlechterung von 37% der Mitarbeitenden der Pilotlabore, Z3d genannte Verschlechterungen von 35% der Mitarbeitenden der V1. Rundungsbedingt können in den Grafiken geringfügige Abweichungen auftreten, wodurch die Ergebnisse nicht immer 100% ergeben.

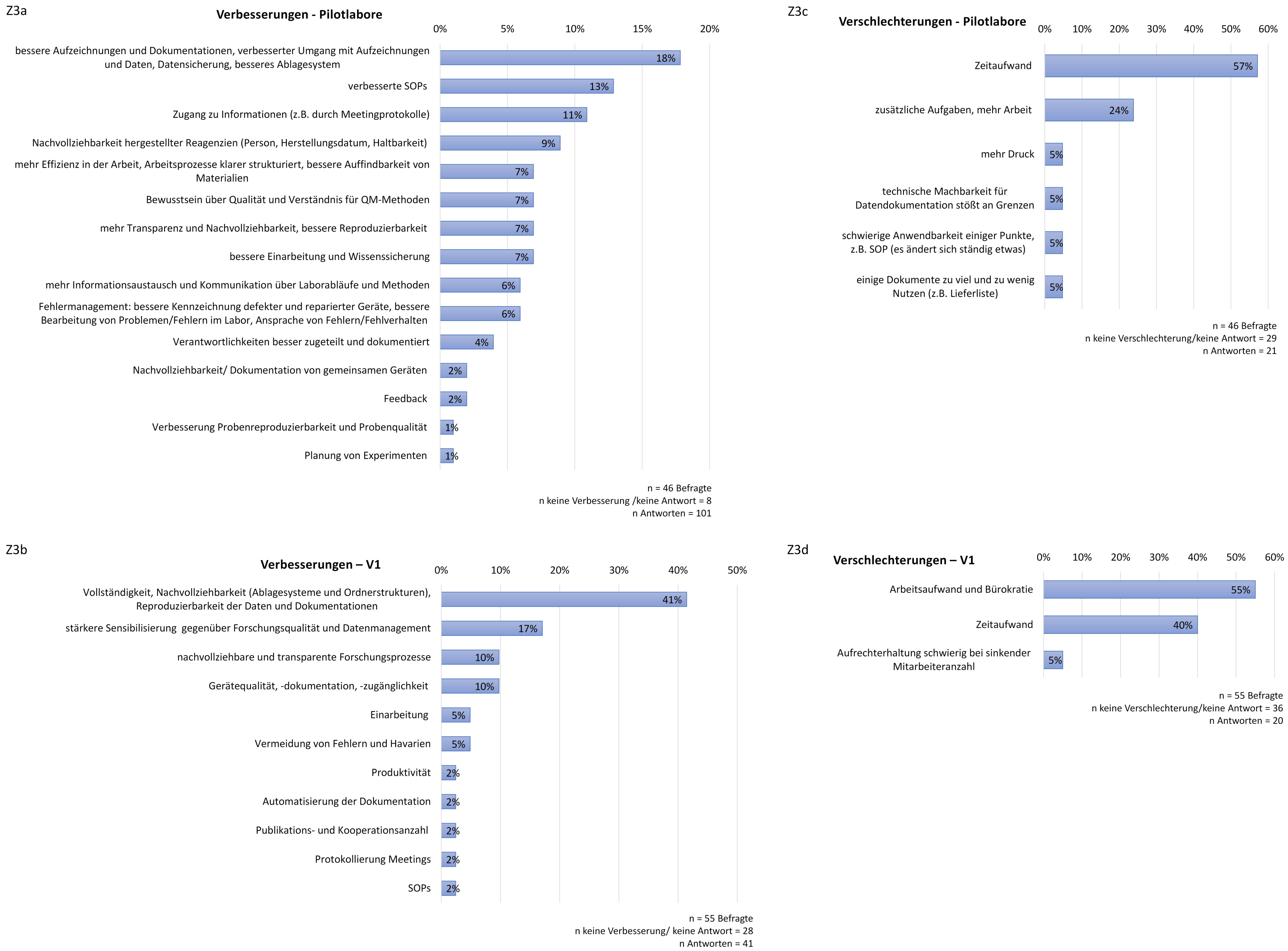

Supplement: Supplementary file 3 — ESM3: Zusatzmaterial 3 [file 103_2026_4191_MOESM3_ESM.pdf]
